# Effectiveness and acceptability of the unified protocol for the transdiagnostic treatment of emotional disorders in people with long COVID-19: Study protocol for a randomized controlled trial

**DOI:** 10.1371/journal.pone.0342908

**Published:** 2026-02-17

**Authors:** Verónica Martínez-Borba, Andrés E. Rodríguez-Márquez, Sara Garcés-Arilla, Óscar Peris-Baquero, María Vicenta Navarro-Haro, Esther del Corral-Beamonte, Jorge Osma

**Affiliations:** 1 Department of Psychology and Sociology, Faculty of Social and Human Sciences, Universidad de Zaragoza, Teruel, Spain; 2 Instituto de Investigación Sanitaria de Aragón, Zaragoza, Spain; 3 Internal Medicine, Hospital Royo Villanova, Zaragoza, Spain; PLOS: Public Library of Science, UNITED KINGDOM OF GREAT BRITAIN AND NORTHERN IRELAND

## Abstract

**Background:**

Long COVID-19 is a medical condition associated with persistent physical, cognitive, and emotional symptoms. Despite its significant impact, there are still few psychological interventions—especially with transdiagnostic approaches— that have been rigorously tested in this population. The aim of the present protocol is to describe a randomized controlled trial to examine the effectiveness and acceptability of the online, group-delivered Unified Protocol (UP) for improving emotional, and cognitive outcomes in adults with long COVID-19. We expect greater improvements in emotional and cognitive outcomes for the UP group compared to controls. Additionally, exploratory analyses will assess changes in neurocognitive performance and hair cortisol/cortisone levels as potential correlates of treatment response.

**Methods:**

90 individuals diagnosed with long COVID-19 will be randomized to an experimental group or a waiting-list control group (1:1 ratio). Participants in the experimental group will receive the UP across 12 online group sessions. Longitudinal assessments (pre-treatment, post-treatment and 3, 6 and 12 months follow-ups) will include psychological (e.g., anxiety and depressive symptoms) and cognitive outcomes (e.g., memory failures). Participants in the experimental group will also complete neuropsychological tests and will provide hair samples for the assessment of cortisol/cortisone levels.

**Data analyses:**

Baseline characteristics will be described using descriptive statistics, and linear mixed-effects models will evaluate the effects of time, group, and their interaction on psychological and cognitive outcomes. Neuropsychological performance and hair cortisol levels will be analyzed over time in the experimental group. Associations between cortisol and psychological or cognitive measures will be explored through correlational analyses.

**Conclusions:**

We expect positive outcomes after the intervention in acceptability and in emotional symptoms and cognitive complaints in individuals living with long COVID-19, the maintenance of the benefits in all follow-ups, and statistically significant changes in favor of the UP condition in comparison with the waiting-list control group. If effective, the UP could provide an accessible and evidence-based psychological treatment for this population, improving the quality of healthcare to individuals with long COVID-19.

**Trial registration:**

clinicatrials.gov (registration identifier: NCT06928480; May 22, 2025).

## Introduction

The World Health Organization (WHO), a global leader in the study and management of public health emergencies, has been committed, since the beginning of the COVID-19 pandemics, to studying not only the acute symptomatology but also the evolution of individuals affected by the SARS-CoV-2 virus. As early as September 2020 the term “post COVID condition” was included in one of the main diagnostic medical tools worldwide, the International Classification of Diseases [ICD-10 code (U09)]. This diagnostic criteria was made by the WHO to distinguish between acute infection, late effects, and prolonged COVID-19 disease [1]. However, the complete definition of this condition was not formally established until October 2021, as stated below:

Post COVID-19 condition occurs in individuals with a history of probable or confirmed SARS CoV-2 infection, usually 3 months from the onset of COVID-19 with symptoms and that last for at least 2 months and cannot be explained by an alternative diagnosis. Common symptoms include fatigue, shortness of breath, cognitive dysfunction but also others and generally have an impact on everyday functioning. Symptoms may be new onset following initial recovery from an acute COVID-19 episode or persist from the initial illness. Symptoms may also fluctuate or relapse over time [2].

Clearly defining the medical profile of patients with long COVID can be challenging, as more than 200 symptoms have been described [3], making it a condition with substantial heterogeneity in its clinical manifestations. A recent systematic review and meta-analysis including 36 studies with long COVID-19 patients and more than 11.000 participants revealed the presence of general (e.g., pain, fatigue, fever, hair fall, skin rash, weight loss), neurological (e.g., headache, cognitive impairment, loss of smell, taste and hearing), cardiopulmonary (e.g., chest pain, sore throat, dyspnea, palpitations and cough), and gastrointestinal symptoms (e.g., poor appetite, diarrhea, nausea, abdominal pain) [4]. Similarly, variations in the prevalence of this condition has been exposed, but currently it has been estimated that 6 in every 100 people who have been infected with COVID-19 develop post COVID-19 (or long covid) condition [2].

Apart from the WHO, other internationally recognized organizations have also acknowledged the relevance of the long COVID-19 condition and the urgency of addressing it promptly. Thus, the European Network of expertise on Long COVID-19 from the European Commission [5], the Center for Disease Control and Prevention (CDC) from the United States [6] or the National Institute for Health and Care Excellence (NICE) from United Kingdom [7] have postulated the need to better prevent and treat long COVID-19 conditions. However, it is also noteworthy that interest has usually focused on the physical recovery of symptoms instead of helping people with long COVID-19 to psychologically adapt to the illness and prevent or treat the emotional disorders that could emerge.

Psychological care for patients with long COVID-19 should not be neglected as numerous studies have shown that emotional suffering is markedly prevalent among these individuals. More precisely, a recent systematic review and meta-analysis including 143 studies and over 7 million participants has revealed that the prevalence of anxiety and depressive symptoms in patients with long COVID-19 is around 23% [8]. This alarming prevalence of emotional symptoms in patients with long COVID-19 has led to increased interest in developing and implementing psychological interventions for COVID-19 populations. However, a recent systematic review [9] have noted that intervention studies have primarily focused on acute COVID-19 patients, relatives and healthcare professionals, and to a lesser extent, on long COVID-19 patients. Furthermore, another recent systematic review [10] specifically examining interventions for individuals with long COVID-19, found that psychological support has not been a primary focus. Of the 31 included studies, only seven evaluated psychological interventions, whereas the remaining studies were centered on neurocognitive rehabilitation, natural supplementation, pharmaceutical treatments or physical rehabilitation programs [10]. A more detailed analysis of these seven psychological interventions shows that only two employed a rigorous methodological design as randomized controlled trial (RCT), and the therapeutic psychological approaches used are highly heterogeneous, including cognitive processing therapy, acceptance and commitment therapy, peer support or cognitive-behavioral therapy (CBT) [10]. Also importantly, although some participants included in the review presented comorbid anxiety and depressive symptoms (see the study by Skilbeck [11]) we also observed a lack of transdiagnostic approaches and a predominance of disorder-specific psychological interventions.

Even when CBT is one the psychological interventions with most accumulated evidence for adults with emotional disorders [12] and also populations with chronic physical conditions [13], focusing on specific cognitive-behavioral interventions for specific emotional disorders may present some important limitations. One major issue with applying disorder‑specific psychological programs — for example, a therapy designed only for depression — is that many patients present comorbidity, especially between anxiety and depressive symptoms [14], which limits the dissemination of single‑disorder treatments. The transdiagnostic approach — as embodied in the Unified Protocol (hereafter UP) — aims to overcome this limitation by targeting shared underlying processes (e.g., emotion dysregulation) across multiple emotional disorders [15]. Different RCT have demonstrated the non-inferiority of the UP as it achieves reductions in anxiety and depressive symptoms comparable to those produced by standard evidence-based single-disorder treatments [16,17]. Also importantly, the UP has demonstrated its usefulness in prevent or treat emotional disorders in individuals with medical health conditions [18]. Given the high rates of comorbidity and overlapping mechanisms in emotional disorders, transdiagnostic treatments like the UP may represent a more parsimonious and flexible solution for individuals with emotional disorders and long COVID-19 than multiple disorder‑specific interventions [19].

It is also important to note that emotional symptoms in long COVID do not appear to occur in isolation but are frequently accompanied by cognitive difficulties. Individuals with long COVID-19 show prevalence rates of cognitive complaints (particularly difficulties in memory and concentration) ranging from approximately 21% to 25%, which are comparable to those reported for anxiety and depressive symptoms (for a meta-analysis, see the study from van der Feltz-Cornelis [20]). Moreover, evidence from long COVID-19 population suggests that greater emotional symptomatology is associated with poorer cognitive performance [21]. Taken together, these findings underscore the importance of developing and evaluating psychological interventions targeting emotional symptoms in individuals with long COVID, in order to examine whether improvements in emotional functioning are accompanied by benefits in cognitive performance, an area that remains largely unexplored in the current literature.

With all this information in mind, the primary aim of this RCT is to assess the effectiveness and acceptability of the UP delivered in an online group format for treating emotional disorders in long COVID-19 patients. We hypothesize that participants in the experimental group will show improvements in psychological and cognitive outcomes. Specifically, we expect that at post-treatment participants in the experimental group will exhibit reductions in depressive and anxiety symptoms, memory failures, dimensions of emotional disorders (neuroticism, depressed mood, somatic anxiety, arousal activation, social anxiety, intrusive cognitions, traumatic re-experiencing and avoidance), difficulties in emotion regulation and perceived stress. In addition, we anticipate increases in quality of life, distress tolerance and positive temperament scores. We expect these improvements to be maintained at follow-up assessments conducted 3, 6 and 12 months after the intervention. We further hypothesize that participants in the experimental group will show greater improvements in psychological and cognitive outcomes compared to those in the control group. Additionally, the study will examine participant acceptability and satisfaction with the UP intervention and the online group format of delivery, with expectations of high satisfaction and adherence rates.

The secondary aim of this study is to explore the evolution of neurological, cognitive and physiological outcomes in a sample of long COVID-19 patients. Longitudinal changes in cognitive impairments (i.e., visuospatial skills, memory, working memory, attention, concentration, language, executive functions, orientation and verbal short-term memory), cortisol and cortisone levels will be measured in the experimental group to investigate potential neurological and physiological correlates of the UP intervention. To the best of our knowledge, no studies have explored the usefulness of the UP in reducing cognitive impairments or cortisol/cortisone levels in long COVID-19 patients. Consequently, this aspect of the study is exploratory in nature and no formal hypotheses are proposed.

## Methods

### Design

This study protocol describes a two-arm parallel superiority RCT (allocation ratio 1:1). The trial has been designed to test the superiority of the experimental treatment (the UP) over the control condition (waiting list control group) in the improvement of depressive and anxiety symptoms, memory failures, dimensions of emotional disorders, emotional dysregulation, perceived stress, quality of life, distress tolerance and positive temperament in long COVID-19 participants.

The randomization sequence will be generated using the online platform randomizer.org to establish the order of group assignment (e.g., participant 1 = control; participant 2 = control; participant 3 = experimental; participant 4 = control; participant 5 = experimental; participant 6 = experimental…participant 90 = control). As participants enroll in the study, they will be assigned to the experimental or control group following this predefined sequence. Since the control group will remain on the waiting list for 12 weeks, participants cannot be blinded to their assignment condition. For the same reason, the researchers will also be aware of participants’ group assignments.

To ensure the transparency and methodological rigor of the study it was registered in clinicatrials.gov (registration identifier: NCT06928480; date of registration: May 22, 2025). Additionally, this study protocol adheres to the Standard Protocol Items: Recommendations for Interventional Trials (SPIRIT checklist [22]; see supporting information S1 Checklist).

### Trial status

Participant recruitment started on May 26, 2025 and is expected to conclude by December 31, 2026. Data will be collected during the recruitment period and will be finalized by the same date. Subsequent data analysis will be carried out once data collection is complete, with results expected to be available in 2027.

### Participants

Participants will be adults residing in the Autonomous Community of Aragon (Spain) who have been diagnosed with long COVID-19 and meet criteria for at least one comorbid emotional disorder. Recruitment will take place at Hospital Royo Villanova from Zaragoza, Spain. An online dissemination campaign will also be conducted through social medial and in collaboration with the Long COVID-19 association from Aragón. Eligibility criteria to participate in the study are reported in Table 1. Participants will not be involved in the design, conduct or reporting the trial.

**Table 1:** Inclusion and exclusion criteria to participate in the randomized controlled trial.

Inclusion criteria	Exclusion criteria
Residing in the Autonomous Community of Aragon (Spain). Being at least 18 years old. Understanding of Spanish. Being diagnosed with long COVID-19: documented SARS-CoV-2 infection and persistence of symptoms beyond 12 weeks after the acute infection. Symptoms of depression (ODSIS≥7) and/or anxiety (OASIS≥8). Meeting the criteria for an emotional disorder diagnosis. Having access to the Internet. Signing the informed consent.	Pre-existing emotional symptoms prior to the acute SARS-CoV-2 infection. Currently receiving psychological treatment. Having a diagnosis of severe mental disorder (e.g., personality disorder, bipolar disorder, etc.). Active suicidal ideation at the time of the assessment. Individuals on psychotropic medication must maintain their dosage throughout the study, unless medically contraindicated.

Using the G*Power software [23], and taking into account the two study conditions, the five assessment points (pre-treatment, post-treatment, and follow-ups at 3, 6, and 12 months), as well as the statistical models planned for data analysis, we calculated a total sample size of 74 participants, achieving a statistical power of 90%, an alpha level of 0.05, and an effect size of 0.30. Considering an anticipated dropout rate of 20%, the required sample size is estimated at 45 participants per condition (total sample size = 90 participants).

### Procedures

All procedures described in this study were approved by the Ethics Committee of the Autonomous Community of Aragón. Changes to this original study protocol (current protocol version number 2; May 2, 2025) will be communicated to the aforementioned Ethics Committee for their approval.

Physicians and nurses working in the long COVID-19 unit will invite patients to participate in the study. Participants who are interested in the study will receive a document containing a QR code to access the online Qualtrics platform, where they can read and electronically sign the informed consent form. After signing the online document, participants will receive a numerical code to preserve their anonymity in subsequent online assessments, and will then complete the Overall Depression Severity and Impairment Scale (ODSIS; [24,25]) and the Overall Anxiety Severity and Impairment Scale (OASIS; [25,26]) to assess the presence of depressive and/or anxiety symptoms that helped the research team to determine whether they meet the initial eligibility criteria.

Participants who do not meet the ODSIS and OASIS criteria will receive an email thanking them for their interest in the study and informing them that they do not meet the initial eligibility criteria. In contrast, participants who meet the cutoff criteria for anxiety and depressive symptoms (ODSIS ≥ 7 and/or OASIS ≥ 8) will be contacted by telephone by the psychologist responsible for conducting the initial clinical interview and the subsequent psychological UP-based program. During this call, the psychologist will confirm that the participant is interested in taking part in the study and agrees to the conditions of the intervention (i.e., the possibility of being assigned to the waiting list control group, participation in the psychological intervention in an online group format, agreement with the assessment schedule, and provision of hair samples for cortisol/cortisone analysis). Once it is confirmed that the participant understands the study’s conditions and procedures, an online session via videoconference will be scheduled to conduct a clinical interview to determine the presence of an emotional disorder.

After confirming the presence of at least one emotional disorder according to the Anxiety and Related Disorders Interview Schedule for DSM 5 (ADIS-5; [27]), participants will complete the online pre-treatment assessment protocol and will then be informed by email of their group assignment (either the experimental or the waiting list control group). Randomization to the experimental or group condition will be conducted by an independent researcher who will consult the pre-generated randomization sequence. The psychologist will also be informed of the assigned group to facilitate the management and organization of the UP-based experimental group intervention.

Participants in the experimental group will be required to complete an in-person additional assessment before they can begin the program. Accordingly, individuals assigned to the experimental group will meet in Zaragoza with a member of the research team to complete the neuropsychological and cognitive tests and to provide a hair sample for subsequent cortisol/cortisone analysis (secondary objective of this study). After these assessment point, participants will receive the UP-based psychological intervention in 12 online group sessions, each lasting 2 hours. Given that long COVID-19 patients often report cognitive difficulties [28] a break of approximately 10 minutes will be scheduled midway through each session. After the intervention, participants will complete the online post-treatment assessment protocol, which will include the measures administered at pre-treatment and a satisfaction questionnaire. Follow-up online assessments will also be conducted at three, six and twelve months after the intervention. At these time points, a group online session will be scheduled to monitor progress and reinforce the emotion regulation skills learned during the UP psychological program. At the three and twelve-month follow-up assessments, participants will repeat the in-person assessment to complete the neuropsychological and cognitive tests and to provide the hair samples for cortisol/cortisone analyses. Participants will receive email reminders for these appointments.

In the case of participants who will be assigned to the waiting list control group, they will remain on the waiting list for 12 weeks and will not receive any psychological intervention. After this waiting period, they will complete the post-treatment assessment protocol and will then be enrolled in the experimental group following the same procedures previously described for this condition.

### Outcomes

**Fig 1:**
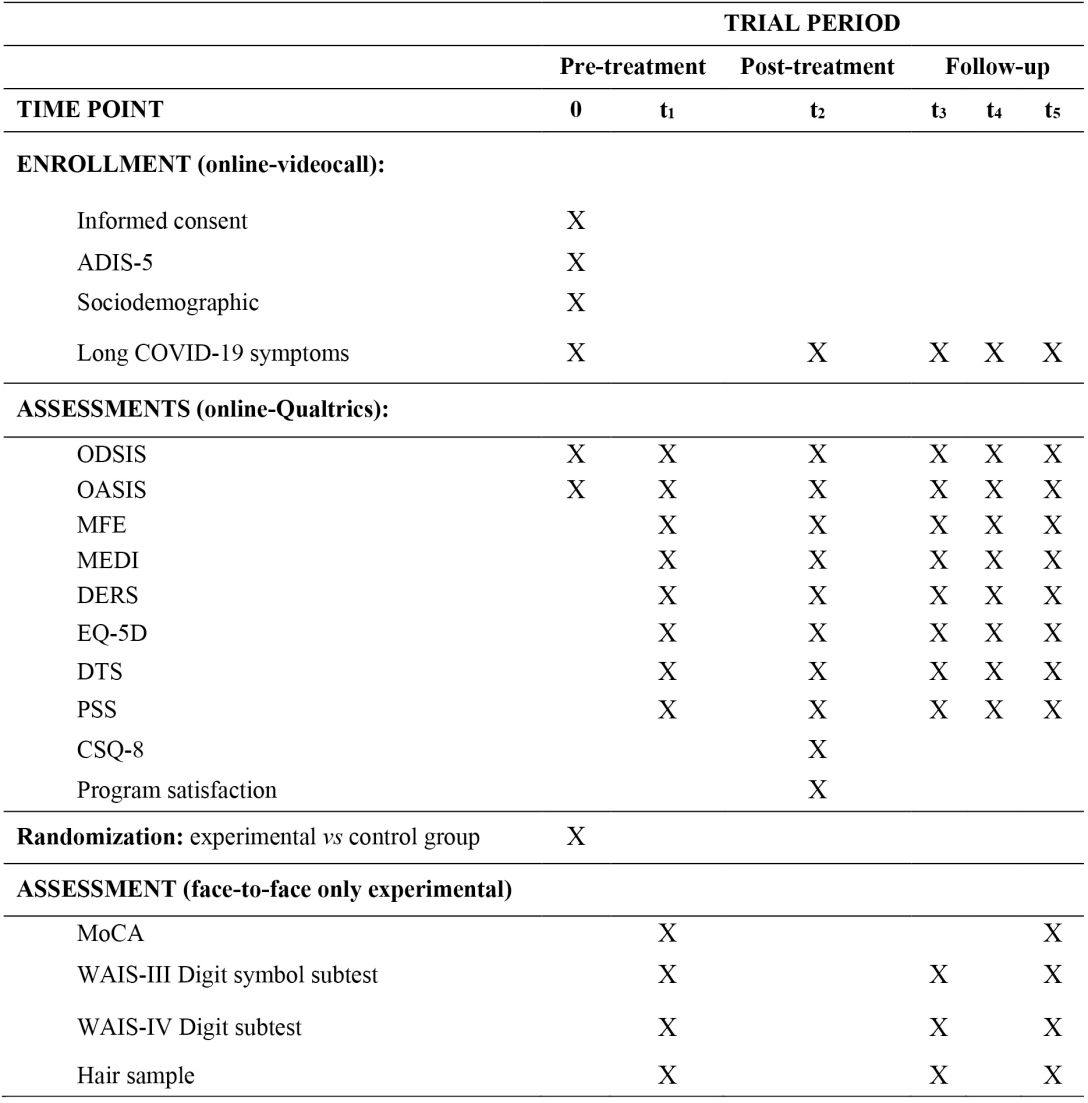
Participant timeline. Note: ADIS-5: Structured interview for anxiety disorders and related disorders; ODSIS: Overall Depression Severity and Interference Scale; OASIS: Overall Anxiety Severity and Interference Scale; MFE: Memory Failures of Everyday; MEDI: Multidimensional Emotional Disorders Inventory; DERS: Emotional Regulation Difficulties Scale; EQ-5D: EuroQol; DTS: Distress Tolerance Scale; PSS; Perceived Stress Scale; CSQ-8: Client Satisfaction Questionnaire; MoCA: Montreal Cognitive Assessment; WAIS-III: Digit Symbol subtest-Wechsler Adult Intelligence Scale-III; WAIS-IV: Digit subtest from Wechsler Adult Intelligence Scale-IV.

As previously explained in the procedure section, the assessment protocol will include evaluations carried out via videocall, as well as in-person sessions and the completion of online surveys through the Qualtrics platform. Following the SPIRIT recommendations [22], Fig 1 presents the questionnaires administered at each time point as well as the administration format. In the following section, we briefly describe the questionnaires that will be administered.

#### Eligibility criteria and sample characteristics: Online videocall assessment.

- Anxiety and Related Disorders Interview Schedule for DSM-5 (ADIS-5) [27]: this interview will serve to determine whether participants meet criteria for an emotional disorder. This interview allows us to assess major depressive disorder, dysthymia, panic disorder, agoraphobia, obsessive-compulsive disorder, generalized anxiety disorder, post-traumatic stress disorder, social anxiety disorder, hypochondria, and adjustment disorders. In our study, participants showing symptoms consistent with anxiety or depressive disorders “not otherwise specified” will also be considered for inclusion in the study.
- Sociodemographics: the research team has developed an *ad hoc* questionnaire to obtain sociodemographic data from participants. Thus, information about sex, age, place of residence, marital status, number of children, employment status, income, lifestyle habits (sleep, smoking, alcohol, physical activity, diet) will be collected at pre-treatment.
- Long COVID-19 questionnaire: participants will be asked about their COVID-19 and long COVID-19 history, including questions regarding the number and dates of COVID-19 infections, the number and dates of COVID-19 vaccine administrations, the date of long COVID-19 diagnosis, and physical symptoms related to COVID-19 (from their first infection and at the present).

#### Primary outcomes: Online survey assessment.

- Overall Depression Severity and Interference Scale (ODSIS) [24,25]: depressive symptoms are assessed across five items evaluating frequency, intensity, severity, and interference of symptoms. Scores range from 0 to 20 points, with higher scores denoting greater depressive symptoms severity. While the Spanish validation of the questionnaire established a cutoff score of 10 points [25], in this study, and similar to previous research [29], the cutoff has been reduced to 7 points to ensure that all participants who may be experiencing depressive symptoms are thoroughly assessed for eligibility and can benefit from the psychological intervention. Thus, ODSIS cut-off was established at approximately half standard deviation below the mean scores obtained in the Spanish validation of this questionnaires (mean=9.87, *SD*=5.14) [25].
- Overall Anxiety Severity and Interference Scale (OASIS) [25,26]: this instrument measures anxiety symptomatology across five items assessing frequency, intensity, severity, and interference of symptoms. Total scores vary from 0 to 20 points; higher values indicate more severe anxiety symptoms. As explained in the section describing the depression questionnaire (ODSIS), in this study the cutoff score for the OASIS has been reduced to 8 points, taking into account the mean and standard deviation reported in the Spanish validation (mean=10.45, SD=4.49) [25]. This threshold corresponds to approximately half standard deviation below the Spanish mean, a criterion that allows participants with subclinical anxiety symptoms to be assessed and, if they met criteria for an emotional disorder, to participate in the study.

#### Secondary outcomes: Online survey assessment.

- Memory Failures of Everyday (MFE) [30,31]: the scale assesses how frequently individuals experience forgetfulness or memory lapses in everyday situations. It comprises 28 items and includes three factors: forgetting activities (i.e., failing to remember tasks or where objects are placed; maximum score: 20), difficulties recognizing places or people (maximum score: 12), and problems with communication monitoring and control (i.e., following a story, forgetting words or repeating stories among others; maximum score: 24). Higher scores in each subfactor indicate greater memory failures.
- Multidimensional Emotional Disorders Inventory (MEDI) [32,33]: this measure includes 49 items designed to assess the transdiagnostic profile of emotional disorders. It covers nine dimensions: neurotic temperament (5 items), positive temperament (5 items), depressed mood (5 items), somatic anxiety (5 items), arousal/activation (5 items), social anxiety (5 items), intrusive cognitions (6 items), traumatic re-experiencing (5 items), and avoidance (8 items). As shown, the number of items per dimension ranges from 5 to 8. Dimension scores are obtained by summing the corresponding item scores, resulting in total scores ranging from 0 to 40, 0 to 48, or 0 to 64, depending on the dimension. Higher scores reflect higher levels of that dimension.
- Difficulties in Emotion Regulation Scale (DERS) [34,35]: this instrument consists of 28 items that assess difficulties in emotion regulation across five subscales: lack of control (9 items), rejection (7 items), interference (4 items), inattention (4 items), and emotional confusion (4 items). It is also possible to obtain a global emotion dysregulation score by summing the scores of all items. Higher scores indicate greater difficulties in regulating emotions.
- EuroQol (EQ-5D) [36,37]: this questionnaire is used to evaluate participants’ health-related quality of life. It includes five items, each assessing one dimension of quality of life (i.e., mobility, self-care, usual activities, pain or discomfort, and anxiety or depression). In addition, this measure includes a visual analogue scale (VAS) on which respondents rate their overall health from 0 to 100. Higher scores on the VAS indicate better perceived health status.
- Distress Tolerance Scale (DTS) [38]: this scale assesses distress tolerance across 15 items, covering the following dimensions: (1) Tolerance (i.e., the perceived ability to withstand emotional distress; 3 items); (2) Appraisal (i.e., the subjective evaluation of distress; 6 items); (3) Absorption (i.e., the extent to which attention is captured by negative emotions; 3 items); and (4) Regulation (i.e., the strategies used to alleviate distress; 3 items). A global distress tolerance score can be obtained, with total scores ranging from 15 to 75. Higher scores indicate greater tolerance to distress.
- Perceived Stress Scale (PSS) [39,40]: this scale includes 14 items that assess the extent to which individuals have experienced stress in their daily lives during the past month. The authors of the Spanish validation proposed a shorter 10-items version of the PSS, which will be used in the present study. Total scores range from 0 to 40, with higher scores reflecting greater levels of perceived stress.
- Adaptation of Client Satisfaction Questionnaire (CSQ-8) [41]: while the original version of this questionnaire is composed of 8 items, in this study 6 of these items will be used to assess participants’ satisfaction with the intervention. These items cover (1) perceived quality of the program, (2) fulfillment of expectation, (3) likelihood of recommending the program, (4) perceived usefulness of the techniques, (5) general satisfaction with the program, and (6) willingness to participate in similar interventions in the future. An additional item will be included to evaluate any discomfort elicited by the intervention.
- Qualitative satisfaction reports: to capture participants’ qualitative feedback, five open-ended questions will be also presented: (1) Are there topics you would like to see included that were not addressed? (2) Is there any content you consider unnecessary and would remove? (3) Was the program duration (12 sessions of 2 hour each) sufficient? (4) How satisfied are you with the online, group format of the program? (5) Please share any additional concerns or comments regarding the program.
- Evaluation questionnaire of the UP modules (*ad hoc*): seven questions will be included in the satisfaction questionnaire to assess the perceived satisfaction with the UP-based psychological intervention. A general question about the usefulness of the UP for improving emotional regulation was first presented. Then six specific items will be shown to evaluate the perceived usefulness of each technique learned during the UP program. Total scores for each item ranges from 0 to 10 with higher scores indicating greater satisfaction with the intervention.

#### Secondary study objective: Face-to-face assessment.

- Montreal Cognitive Assessment (MoCA) [42]: this neurological test will be used to obtain a score of the overall level of cognitive abilities. It assesses several cognitive domains, including visuospatial skills, memory, working memory, attention, concentration, language, executive functions, and orientation. The maximum total score is 30 points. Scores between 26 and 30 may be interpreted as indicative of normal cognitive functioning, whereas scores of 25 or below suggests the presence of cognitive impairment.
- Digit Symbol subtest-Wechsler Adult Intelligence Scale-III (WAIS-III) [43]: this cognitive test assesses psychomotor speed, sustained attention, and incidental learning and is a paper-and-pencil subtest of the Wechsler Adult Intelligence Scale–III. Participants are required to match digits (1-9) with their corresponding symbols as quickly as possible within a 120-second time limit. The number of correctly matched symbols constitutes the matching score (maximum score = 133). Immediately afterwards, incidental learning is assessed through an untimed cued-recall task (maximum score = 18) and an untimed free-recall task without digit cues (maximum score = 9).
- Digit subtest from Wechsler Adult Intelligence Scale-IV (WAIS-IV) [44]: this subtest assesses verbal short-term and working memory, and it is administered orally. In the Digit Span Forward task, participants must repeat sequences of numbers in the same order, which provides an indication of their verbal short-term memory. In the Digit Span Backward task, participants reproduce number sequences in reverse order to assess verbal working memory. Memory capacity is defined as the maximum span length, which is the longest sequence that can be correctly recalled. The maximum span is 9 for the Digit Span Forward task and 8 for the Digit Span Backward task. Two trials are administered at each sequence length. Additionally, performance can be expressed as a raw score, which is calculated by adding together the number of sequences correctly recalled, with a maximum raw score of 16 for both tasks. Higher scores indicate better verbal short-term and working memory performance.
- Cortisol and cortisone: hair samples will be collected from the posterior vertex or nape area, where hair growth is continuous and less exposed to external contaminants. This sampling site is recommended due to its lower intra-individual variability and greater representativeness of long-term systemic hormone exposure. A small section of hair will be tied with thread near the cut end to identify the portion closest to the scalp during analysis and to prevent hair loss. Using clean scissors, the hair will be cut as close as possible to the scalp, obtaining a segment approximately 3 cm in length. This length allows for the assessment of cortisol and cortisone levels accumulated over the past three months, based on an average hair growth rate of 1 cm per month.

### Intervention

#### Experimental group.

Participants assigned to the experimental condition will receive the UP in group format. The UP is a transdiagnostic CBT intervention composed of eight modules designed to train participants in emotion regulation skills [45]. In this study these eight modules will be distributed to be applied through 12 online sessions (videocalls). The distribution of the sessions can be consulted in Table 2. The sessions will be conducted by a research team trained in the UP (Level II certification) and supervised by a clinician with Level III UP certification. In addition, treatment fidelity to the UP will be monitored throughout the course of the sessions.

**Table 2:** Distribution of the UP sessions.

UP modules	Sessions
1. Setting goals and maintaining motivation	1
2. Understanding emotions	2 - 3
*3. Mindful emotion awareness*	4 - 5
*4. Flexible thinking*	6 - 7
*5. Emotional behaviors*	8
*6. Facing physical sensations*	9
*7. Emotional exposures*	10 - 11
8. Relapse prevention	12

Note: the five core UP modules have been indicated in italics.

The first session will serve to introduce the program’s characteristics, to present the research team members (therapist and co-therapist) and provide an opportunity for participants to get to know each other. After this introduction, the content of the session (i.e., setting goals and maintaining motivation) will be presented. Subsequent sessions (i.e., from the second to the twelve session) will follow the same structure: during the first hour, exercises from the previous week will be reviewed. The second hour will include the introduction of the new module’s content, the practice of the corresponding module exercises, and the assignment of the exercises to be completed during the week. After each module (at the end of sessions 1, 3, 5, 7, 8, 9, 11, and 12), the true/false questions proposed in the UP will be administered to ensure that participants have correctly acquired the basic knowledge of the module.

#### Control group.

Individuals assigned to the control condition will remain on a waiting list for the 12 weeks it takes for the experimental group to receive the intervention. During this period of time, participants who are receiving follow-up for their physical health at any healthcare center may continue attending these appointments (i.e., regular visits with doctors, physicians, nurses, etc. to monitor the physical symptoms of long COVID-19). However, they will be asked not to initiate any additional psychological treatment until they have completed their participation in the study. For ethical reasons, once the post-treatment (t_2_) has been completed, participants in the control group will be offered the opportunity to join the experimental group.

Participants in both groups will be informed, both in the informed consent and also during the initial appointment with the research team, that they may withdraw from the study at any time. Likewise, the research team may recommend discontinuation if a worsening of symptoms is observed or if participants did not meet with the required procedures (i.e., attending to the sessions, responding questionnaires, providing hair samples, etc.)

### Data analysis

First, descriptive statistics of clinical psychological diagnosis, sociodemographic, and long COVID-19 symptoms will be explored to characterize the sample at baseline. Means and standard deviations for psychological measures (i.e., ODSIS, OASIS, etc.) at pre-treatment will also be calculated to provide a baseline description of our sample.

Second, the main effect of time, group and time x group interaction will be analyzed. Several linear mixed-effects models will be employed for each dependent variable to be analyzed (i.e., primary outcomes such as anxiety and depressive symptoms; secondary outcomes such as memory failures, emotional regulation difficulties, etc.). For each of these models, Time (within-subject variable; T1 vs. T2 vs. T3 vs. T4 vs. T5) and Experimental Group (between-subject variable; Experimental vs. Waitlist) will be included as fixed effects. Random slopes for participants will also be considered and included as random effects in the model. The models will have the following structure: [Dependent Variable ~ Time × Experimental Group + (1|Participant)].

In participants from the experimental group, descriptive analyses will also be conducted to assess their neuropsychological and cognitive status, as well as stress levels (hair cortisol and cortisone), prior to starting the intervention. Repeated measures ANOVAs with time as the within-subject factor will be performed to determine whether changes occur in these variables over time (i.e., after the intervention and at 3 and 12-months follow up). Exploratory correlational analyses will be conducted to examine associations between hormonal levels and other dependent variables. Subsequently, multiple linear regression analyses will be performed to determine which hormonal variables significantly predict subjective cognitive complaints, objective cognitive performance, and emotional variables, while controlling for relevant covariates (e.g., age and educational level).

Satisfaction with the intervention will be summarized using means and standard deviations derived from the satisfaction questionnaire. Additionally, adherence to the intervention will be calculated as the proportion of attended sessions relative to those scheduled.

Analyses will be conducted using the lme4 package (version lme4_1.1–13; [46]) in R statistical software (version 4.1.0; [47]) and SPSS v25.0 [48].

## Discussion

The present study protocol describes a RCT exploring the efficacy of a transdiagnostic psychological intervention, the UP, in reducing the emotional and cognitive burden of long COVID-19 compared with a waiting list control group. The experimental group receiving the UP is expected to obtain better results in the psychological and cognitive outcomes after the intervention. Previous studies have indicated that long COVID-19 patients experience both emotional and cognitive complaints [4]. Thus, the primary and secondary outcomes of our study include measures of depression, anxiety, emotional disorders' dimensions, emotion regulation difficulties, quality of life, distress tolerance and perceived stress but also indicators of memory failures. Also importantly, in this study it is proposed a secondary objective to explore the evolution of neurological, cognitive and physiological outcomes before and after the application of the UP. Potential implications for clinical practice and research as well as innovations derived from our study are described below.

The UP has demonstrated robust efficacy in reducing anxiety and depressive symptoms in diverse clinical populations [16,17] also in adults suffering chronic medical conditions [18]. However, previous studies have not explored the potential use of the UP to address cognitive impairments. In this regard, in the systematic review by Osma et al. [18], which focuses on medical health conditions, it is observed that none of the studies applying the UP have incorporated neurocognitive assessments or biological markers of stress as an outcome. To the best of our knowledge, only one ongoing study that will deliver the UP in cancer survivors includes cognitive functioning in their assessment protocol [49], but it does not assess physiological correlates such as cortisol or cortisone levels. This gap is particularly relevant in the context of long COVID-19, where anxiety and depressive symptoms have been linked to dysregulation of stress-response systems, especially the hypothalamic–pituitary–adrenal (HPA) axis and alterations in cortisol regulation (for a review, see Narayanan et al. [50]). In general, sustained dysregulation of the HPA axis has been proposed to contribute to functional alterations in brain regions involved in cognitive functioning, including the hippocampus and prefrontal cortex [51]. As an innovative element, the present study proposes a comprehensive evaluation of the UP in adults with long COVID-19, incorporating not only traditional emotional outcomes but also cognitive performance measures (e.g., memory, attention, concentration, executive functions). In addition, hair samples will be collected to assess objective changes in stress levels through cortisol/cortisone measurements. This multifaceted approach represents a novel extension of UP research, addressing both psychological and physiological dimensions of long COVID-19 conditions, and allowing for a better understanding of the broader effects of transdiagnostic interventions in people suffering chronic medical conditions.

Transdiagnostic psychological interventions to improve emotion regulation skills may play a fundamental role in individuals suffering long COVID-19, with the potential to enhance clinical practice by providing clinicians with effective tools to address the emotional and functional challenges faced by this population. Long COVID-19 patients usually experience physical and emotional symptoms that may interfere with their personal goals and daily activities [52]. The UP may help to address this issue because it trains in core emotional regulation skills. Previous studies have revealed that the implementation of these skills in their daily lives may have the potential to reduce not only anxiety and depressive symptoms but also to help patients to tolerate the discomfort generated by unpleasant emotions [53]. It seems that by learning to tolerate distress and engage in goal-oriented behaviors despite physical and emotional challenges, participants may slowly recover a sense of normality. This idea is supported by preliminary findings in long COVID-19 patients, where most therapeutic objectives were achieved over the course of an UP-based intervention [29].

The provision of the psychological intervention in an online group format constitutes also an innovative and cost-effective approach that will also help to overcome practical and clinical challenges found in long COVID-19 patients. Previous studies have indicated that long COVID-19 patients often report fatigue, muscle paint, general pain and joint pain [4] which may impede travelling to onsite healthcare centers to receive in-person attendance. Thus, online sessions will increase accessibility to psychological care in long COVID-19 populations. As for the group format of delivery it may facilitate peer support and shared learning experiences in health conditions [54]. On the other hand, many individuals with long COVID-19 often report feelings of loneliness, stigmatization and discrimination [55–58] and the group format may provide an opportunity to normalize and validate symptoms and to reduce social isolation. In fact, a recent systematic review indicated that people with long COVID-19 frequently use social media forums and online groups to connect with peers who have similar experiences [59]. Also importantly, the program has been designed considering the cognitive and physical limitations of long COVID-19 individuals, including scheduled breaks to help them to participate fully with the sessions. We expect that these patient-focused adaptations will help to facilitate engagement throughout the program, which should in turn contribute to high levels of satisfaction with the intervention. Taken together, these features not only improve accessibility and engagement but also have important clinical implications, as they provide a patient-centered model of psychological care that can be readily implemented in healthcare settings to address the emotional and social needs of long COVID-19 patients.

Despite the results of this study may have important clinical implications, we need to also acknowledge certain limitations. First, participants and researchers cannot be blinded to the intervention assignment, which may introduce expectancy effects. To minimize this potential bias, online assessments will be conducted so that the researchers are not involved in evaluating the efficacy of the intervention. Second, although sample size has been estimated to be sufficient to detect moderate effects, it may limit generalizability to broader populations or to patients with higher or lower long COVID-19 severities. Third, digital literacy of potential participants may interfere or even impede their participation in the study. For instance, participants may present difficulties to engage with the online platform to respond to the psychological assessments or they may find difficulties to engage in the online sessions through videocalls. These potential technology-related challenges will be partially mitigated by the support of the therapist who will explain the procedures for completing the assessment protocol and engaging in the online sessions. Additionally, satisfaction questions regarding the online format will also be included to capture participants’ opinion about the intervention and to evaluate whether these issues have been adequately addressed. Related with participants’ interest in this study, it is possible that some participants may show reluctance when asked to provide a hair sample. To minimize this potential concern, the research team will explain to participants that the hair sample consists of collecting only 5–6 strands from the back of the head and it will not affect their physical appearance. Finally, another potential limitation is that, for ethical reasons, participants in the control group will be offered the opportunity to join in the experimental group after completing the post-treatment assessment. Consequently, only the experimental group will be available for follow-up assessments, and comparisons between groups will be limited to the post-treatment time point.

In conclusion, this trial seeks to address an important gap in mental health care for long COVID-19 populations. By implementing a flexible, group-based, and online transdiagnostic intervention, the study has the potential to improve emotional well-being, enhance emotion regulation skills, reduce cognitive complaints and provide insights for both clinical practice and public health strategies. If our results proved the effectiveness of the UP delivered in an online group format it could offer a valuable, scalable, accessible, and evidence-based psychological intervention to address the psychological needs of long COVID-19 patients.

## Supporting information

S1 Checklist SPIRIT 2025 checklist of items to address in a randomized trial protocol. (PDF)

S1 File Research project report for ethics committee (Spanish). (PDF)

S2 File Research project report for ethics committee (English). (PDF)
