# Supplementary material for: Effectiveness and acceptability of the unified protocol for the transdiagnostic treatment of emotional disorders in people with long COVID-19: Study protocol for a randomized controlled trial
Source: PLoS One. 2026 Feb 17;21(2):e0342908. doi: 10.1371/journal.pone.0342908 (PMC12912558; doi:10.1371/journal.pone.0342908)
Supplement: S1 File — (PDF) [file pone.0342908.s002.pdf]

S2- Supporting information 2

MEMORIA DE PROYECTO DE INVESTIGACIÓN PARA EL CEICA

**TÍTULO**	Eficacia y Aceptabilidad del Protocolo Unificado para el Tratamiento Transdiagnóstico de los Trastornos Emocionales en Personas con COVID-19 persistente
**VERSIÓN Y FECHA**	Versión 2.0 del 02 de mayo de 2025
**CENTRO DONDE SE REALIZA EL ESTUDIO**	Hospital Royo Villanova
**INVESTIGADOR PRINCIPAL DEL PROYECTO**	
**NOMBRE Y APELLIDOS***	Jorge Osma López
**E-MAIL***	protolocolounificadocovid19@gmail.com
**PUESTO DE TRABAJO**	Profesor Titular
**SERVICIO/DEPARTAMENTO**	Departamento de Psicología y Sociología de la Universidad de Zaragoza
**CENTRO/ FACULTAD- UNIVERSIDAD/ OTRO**	Facultad de Ciencias Sociales y Humanas / Campus Teruel
**PROMOTOR (Imprescindible para ensayos clínicos y estudios observacionales con medicamentos)**	
**IDENTIFICACIÓN EMPRESA/ NOMBRE Y APELLIDOS***	Universidad de Zaragoza
**E-MAIL***	gesinves@unizar.es
**CONTACTO DE LA PERSONA ENCARGADA DE GESTIONAR LA SOLICITUD** *Si lo desea, añada los datos de contacto de la persona encargada de gestionar los detalles de la solicitud*	
**NOMBRE Y APELLIDOS**	Verónica Martínez Borba
**E-MAIL**	v.martinez@unizar.es

Los datos de carácter personal que pudieran constar en esta comunicación serán incorporados al sistema de tratamiento del que es responsable el Instituto Aragonés de Ciencias de la Salud (IACS). Los datos serán tratados para la gestión y seguimiento de los estudios evaluados por el CEICA. Los datos serán suprimidos cuando se haya dado respuesta a la gestión y/o tramitación de la solicitud y hayan dejado de ser necesarios. Tiene derecho a acceder, rectificar y suprimir los datos, así como los demás derechos que le otorga la normativa de protección de datos ante el IACS, con domicilio en el Centro de Investigación Biomédica de Aragón. Avda. San Juan Bosco, nº 13, 500009, Zaragoza o solicitándolo a través del mail protecciondedatos.iacs@aragon.es.

GLOSARIO

- Anonimización: proceso por el cual deja de ser posible establecer por medios razonables el nexo entre un dato y el sujeto al que se refiere. Es aplicable también a la muestra biológica.
- BIGAN: plataforma de big data sanitario (gestionada por el IACS) que permite acceder a los datos del Sistema Aragonés de Salud de forma seudonimidaza para su uso en gestión e investigación.
- Biobanco: establecimiento público o privado, sin ánimo de lucro, que acoge una o varias colecciones de muestras biológicas de origen humano con fines de investigación biomédica, organizadas como una unidad técnica con criterios de calidad, orden y destino.
- Centro: Institución donde se realiza un estudio (hospital, centro de salud, residencia, facultad, clínica privada colegio, etc). **En caso de duda se debe hacer referencia al lugar de donde proceden los participantes** (hospital, colegio, club deportivo, etc)
- Compromiso de Confidencialidad: Documento que, de forma obligatoria, deben firmar los alumnos y residentes que desarrollan alguna actividad en el sistema público de salud (modelo establecido en la Orden SSI/81/2017).
- Consentimiento informado: manifestación de la voluntad libre y consciente válidamente emitida por una persona capaz, o por su representante autorizado, precedida de la información adecuada.
- Dictamen del CEICA: documento que acredita que el CEICA ha evaluado un proyecto de investigación y que dicho proyecto cumple con las normas legales aplicables y los criterios éticos.
- Encuesta: recogida de información en soporte físico o digital, con o sin interacción directa con el sujeto fuente.
- Entrevista: recogida de información con interacción directa con el sujeto fuente, mediante respuestas verbales.
- Equipo de investigación: Conjunto de investigadores que realizan de forma conjunta un proyecto concreto.
- Fuente primaria de datos: Cuando los datos se recogen directamente del participante en el estudio y con motivo del estudio.
- Fuente secundaria de datos: Cuando se utilizan para el estudio datos ya recogidos (y registrados, por tanto) que se obtuvieron con una finalidad diferente al estudio (asistencial, docente, etc.).
- Grupo de investigación: Conjunto de investigadores con una trayectoria común (publicaciones, financiación) dirigidos o coordinados por un Investigador Principal, agrupados en torno a una temática de investigación y no necesariamente por su vinculación asistencial o departamental.
- Hoja de información al participante: Documento por el que se informa a los potenciales participantes de la naturaleza del estudio, para que puedan otorgar su consentimiento informado.
- Intervención (estudio de intervención): cualquier actuación que se vaya a realizar sobre una persona debido a su participación en un estudio (puede ser un tratamiento farmacológico, fisioterapéutico, una intervención educativa, conductual, psicológica).
- Investigador principal: Investigador que lidera el proyecto y se hace responsable de su diseño, realización y difusión de los resultados. Si el estudio es multicéntrico debe haber un investigador principal en cada centro que se responsabilice de los pacientes, datos y/o muestras.
- Muestra biológica: cualquier material biológico de origen humano susceptible de conservación y que pueda albergar información sobre la dotación genética característica de una persona
- Práctica clínica habitual: Procedimientos que se llevan a cabo con motivos puramente asistenciales, de forma independiente de la participación o no de una persona en un estudio de investigación.
- Proyecto de investigación: procedimiento científico destinado a recabar información y formular hipótesis sobre un determinado fenómeno social o científico.

- Promotor: individuo, empresa, institución u organización responsable de iniciar, gestionar, organizar y financiar un estudio.
- Seudonimización: el tratamiento de datos personales de manera tal que ya no puedan atribuirse a un interesado sin utilizar información adicional, siempre que dicha información adicional figure por separado y esté sujeta a medidas técnicas y organizativas destinadas a garantizar que los datos personales no se atribuyan a una persona física identificada o identificable.

• **ALCANCE Y FINANCIACIÓN DEL PROYECTO**	
¿Es un proyecto multicéntrico? Sí ☐ No ☒	En caso afirmativo, se debe presentar el listado de centros completo y rellenar un compromiso del equipo investigador **por cada centro de Aragón** (ver anexo I)
¿Dispone de financiación específica para el estudio? Sí ☒ No ☐	En **todos los casos** se debe rellenar el anexo II: autorización uso de recursos. Además, en caso afirmativo, se debe presentar el presupuesto y la fuente de financiación.

• **CARACTERÍSTICAS DEL ESTUDIO**	
**2.1 ¿Se trata de una investigación con medicamentos?** Sí ☐ No ☒	
En caso afirmativo, escoja una opción:<br><br>1) ☐ Se trata de un estudio observacional respecto al tratamiento con medicamentos (EOM)<br>En este caso, especificar:<br>☐ Recogida de datos prospectivos ☐ Recogida de datos retrospectivos ☐ Recogida transversal de datos<br><br>2) ☐ Se trata de un estudio de intervención: ensayo clínico con medicamentos<br>En este caso se debe presentar según instrucciones de la AEMPS (https://www.aemps.gob.es/medicamentos-de-uso-humano/investigacionclinica_medicamentos/ensayosclinicos/#n-espanola)	
**2.2 ¿Se trata de una investigación con productos sanitarios o dispositivos médicos?** Sí ☐ No ☒	
En caso afirmativo, escoja una opción:<br><br>1) ☐ Se trata de un estudio observacional respecto al uso del producto sanitario<br><br>2) ☐ Se trata de un estudio de intervención: ensayo clínico con productos sanitarios<br>En este caso se debe presentar según PNT del CEICA para este tipo de estudios (https://www.iacs.es/investigacion/comite-de-etica-de-la-investigacion-de-aragon-ceica/)	
**2.3 ¿Se trata de una investigación con procedimientos invasivos?**<br>(Definición: toda intervención realizada con fines de investigación que implique un riesgo físico o psíquico para el participante).<br><br>En caso afirmativo se debe contratar una póliza de seguros o justificar riesgo mínimo	Sí ☐ No ☒
**2.4 ¿En la investigación se incluyen menores de edad o personas incapaces de dar su consentimiento?**<br><br>En caso afirmativo se debe presentar un documento de información y consentimiento informado dirigida al tutor/representante legal/familiar y otro dirigido al menor de edad (adaptado a su capacidad). Revisar plantilla CEICA.	Sí ☐ No ☒

2. CARACTERÍSTICAS DEL ESTUDIO	
**2.5 ¿Se utilizan muestras biológicas en el estudio?**	Sí ☒ No ☐
En caso afirmativo, escoja una o varias opciones:<br><br>1) ☐ Se utilizan muestras de excedentes asistenciales con consentimiento para el proyecto<br>Se debe presentar el documento de información y consentimiento (plantilla CEICA)<br><br>2) ☐ Se utilizan muestras de excedentes asistenciales sin consentimiento<br>Se debe justificar adecuadamente en el apartado de aspectos éticos (art. 58.2 Ley 14/2007)<br><br>3) ☒ Se recogen muestras específicamente para este estudio<br>Se debe presentar el documento de información y consentimiento (plantilla CEICA)<br><br>4) ☐ Se crea una colección de muestras privada<br>Para la creación de una nueva colección, presentar la documentación necesaria para su evaluación e indicar nº de registro (nº_____________) (ver web CEICA)<br><br>5) ☐ Se utilizan muestras ya recogidas en una colección de muestras privada<br>En este caso, identificar nº de colección y responsable:______________________<br><br>6) ☐ Se solicitan muestras a un Biobanco autorizado. En este caso, identificar el Biobanco:_________<br>Se debe presentar la solicitud al biobanco	
**2.6 ¿Se realizan análisis genéticos?**	Sí ☐ No ☒
**2.7 ¿Se utilizan embriones, células embrionarias humanas, células o tejidos fetales humanos o bien células humanas pluripotentes obtenidas mediante reprogramación celular?**<br>Debe contactar con el IACS o con la institución responsable para posteriores autorizaciones	Sí ☐ No ☒

3. TRATAMIENTO DE DATOS PERSONALES

Se considera **Dato personal** cualquier dato (edad, sexo) o cualquier información (numérica, alfabética, gráfica, acústica) sobre una persona física identificada o identificable; se considerará identificable toda persona cuya identidad pueda determinarse, directa o indirectamente (es decir, que no se haya anonimizado de forma irreversible en origen)

**3.1 ¿Se recogen o tratan datos de carácter personal en el estudio?**	Sí ☒ No ☐

En caso afirmativo, marca la opción aplicable:

☒Se solicita el consentimiento informado del interesado
Presentar el documento de información y consentimiento (plantilla CEICA)

☐Se utilizan datos obtenidos con otra finalidad que han sido seudonimizados (por ej. Historia clínica, otra investigación, otros registros) conforme a la D.A. 17 de la Ley Orgánica 3/2018.

☐Otros. Especificar:

Marque las **categorías** a las que pertenecen los datos recogidos:

☒ **datos identificativos** (Nombre, dirección, email, DNI, nº Historia clínica, teléfono, firma, IP, geolocalización, imagen/voz, otros)
☒ **datos personales:** fecha nacimiento, lugar de nacimiento, nombre padres, lugar de trabajo, datos económicos, sexo, estado civil, hijos, titulación académica, otros.
☒ **datos de opinión**
☒ **datos especialmente sensibles**: salud, etnia, religión, opinión política, vida u orientación sexual, afiliación sindical, necesidades educativas especiales

3.2 Si los datos se recogen directamente del interesado (fuente primaria), especificar el procedimiento (Ejemplo: entrevista, encuesta en papel, correo electrónico, teléfono, aplicaciones web, …)

Los datos del presente proyecto se recogen a través de dos vías:

- Entrevista online: se realizará mediante la plataforma Google Meet, con una duración estimada entre 60 y 90 minutos. Su principal objetivo será verificar que la persona participante cumple con los criterios de inclusión establecidos para el estudio.
- Cuestionario online: se administrarán a través de la plataforma Qualtrics (https://www.qualtrics.com/support/es/survey-platform/getting-started/qualtrics-gdpr-compliance/#About), garantizando el cumplimiento de la normativa de protección de datos. Las personas recibirán un correo electrónico con un enlace que les permitirá acceder directamente al cuestionario o encuesta correspondiente.
- Cuestionarios en papel: que se administran por parte de un miembro del equipo de investigación en las sesiones presenciales de toma de muestras de cabello del grupo experimental.

3.3 Si no se recogen directamente del interesado (fuente secundaria), marque la opción y especifique:

☐ Se recogen datos de un registro ya existente (ej. Historia Clínica) **con el consentimiento** del interesado.

☐ Se reutilizan datos de otra investigación similar, para la cual se obtuvo el consentimiento del interesado. Se debe presentar permiso del responsable de los datos, el modelo de consentimiento con el que fueron obtenidos y el compromiso de utilización de los datos seudonimizados (ver en la web).

☐ Se utilizan datos obtenidos con otro fin y **sin consentimiento para investigación** (por ej, Historia clínica u otro registro)

En este caso, indicar:

☐ El investigador (si es personal del centro) accede directamente a la historia clínica. Presentar permiso del responsable de los datos (si se trata de la historia clínica, se debe presentar la autorización de la dirección para el acceso a datos para este estudio)

☐ El investigador recibe los datos ya seudonimizados Presentar el compromiso de utilización de los datos seudonimizados (ver en la web).

Nota: **los investigadores que no tengan relación laboral con el hospital/centro** no tienen acceso a la historia clínica, por lo que SIEMPRE que se use esta fuente deben obtener los datos seudonimizados.

En todos los casos, explicar: origen de los datos, responsable de los mismos

☐ Datos procedentes de BIGAN

Presentar informe de la Unidad de Biocomputación (link a la solicitud) y el compromiso de utilización de los datos seudonimizados (ver en la web)

3.4 Una vez obtenida la información y los datos ¿cómo se garantiza la privacidad de los participantes?

☐ Sólo se utilizan datos agregados (es decir, datos que corresponden a grupos de personas y no a cada una de esas personas)

☐ Los datos se anonimizan (los datos no pueden asociarse a una persona identificada o identificable por haberse destruido el nexo irreversiblemente con toda información que identifique al sujeto)

☒ los datos se seudonimizan o codifican (se sustituyen los identificadores directos por un código/seudónimo que sólo conoce el equipo investigador)

Explicar cómo y quién realiza la medida adoptada:

Cada persona participante recibirá un código numérico tras la firma del consentimiento informado. Este código será el que introducirán en las evaluaciones online y en papel con el fin de garantizar que su identidad permanece protegida.

3.5 Plazo de conservación de los datos: especificar fecha de destrucción (al menos mes y año):

Se conservarán los datos hasta la publicación de los trabajos científicos (aproximadamente hasta el 31 de diciembre de 2030).

Generalmente se considera adecuado conservar hasta la fecha de publicación, si no, se debe justificar

3.6 Encargados del tratamiento (no cumplimentar en caso de datos anónimos)

Se entiende por **encargado de tratamiento** a toda persona física o jurídica, autoridad pública, servicio u otro organismo que trate datos personales, ajeno al responsable del tratamiento (investigador).

Si un tercero (ajeno a la institución) trata datos del proyecto, será necesario firmar un **contrato de encargado de tratamiento**. Un modelo se puede descargar de https://seguridad.salud.aragon.es/plantillas/

Indicar qué personas van a tratar los datos recogidos, especificando quién tendrá acceso a los datos identificativos:

Investigador principal: Jorge Osma López
Miembro del equipo: Verónica Martínez Borba
Miembro del equipo: Sara Garcés Arilla
Miembro del equipo: Andrés E Rodríguez Márquez

¿Todas las personas autorizadas a tratar los datos están sujetas a un acuerdo de confidencialidad firmado con el centro?

Sí ☒ No ☐

3.7 ¿Se van a ceder datos a terceros? Sí ☐ No ☒

En caso de cesión se debe especificar:

- los datos cedidos son: ☐ identificados, ☐ seudonimizados, ☐ anonimizados

- a quién se ceden:

- qué datos se ceden:

- con qué finalidad:

- Explique cómo se seudonimizan o se anonimizan los datos:

- Si hay transferencias internacionales: especificar empresa y país (en este caso, se debe solicitar el consentimiento expreso del participante para esta cesión)

TRATAMIENTO DE DATOS PERSONALES	
**3.8 ¿Se van a realizar grabaciones (audio/vídeo)?**<br>Recuerde que se necesita consentimiento expreso del interesado para realizar grabaciones, esta información se debe incluir en el documento de consentimiento	Sí ☐ No ☒

En caso de grabación, se debe especificar:

- Dónde se van a conservar, quién tiene acceso y las medidas de seguridad a aplicar:

- Plazo de conservación de las grabaciones:

- con qué finalidad:

- si se utilizan aplicaciones informáticas o almacenamiento en la "nube", se debe indicar quién es el proveedor de servicios y donde está su residencia legal, así como el enlace a su política de privacidad:

3.9 Medidas de seguridad de la información: descripción de los sistemas informáticos que se van a utilizar

Se recuerda que los servidores que contengan datos personales deben estar ubicados en el territorio de la UE (RDL 14/2019)

- Sistema en el que se van a guardar los datos (ordenador personal, servidores corporativos, empresa u organismo externo, proveedor de servicios en la "nube", etc.)

Los datos del estudio serán almacenados en un fichero creado con el software IBM SPSS, el cual se resguardará en un disco duro interno dentro de los servidores de la Universidad de Zaragoza. La identificación del equipo utilizado para este propósito corresponderá al Despacho 26 de la Facultad de Ciencias Sociales y Humanas de Teruel, con el número de referencia 259116.

- Aplicativos que se van a utilizar para el tratamiento de datos (excel, spss, etc.)

Para el análisis e introducción de datos cuantitativos se utilizará el paquete estadístico IBM SPSS Statistics versión 22.0 para Windows (IBM Corp., 2013). Los datos recogidos para el estudio estarán identificados mediante un código numérico y sólo el personal investigador en el estudio tendrá acceso a ellos y serán almacenados en los dispositivos de la Universidad de Zaragoza.

- Si se utilizan aplicaciones informáticas online o almacenamiento en la "nube", se debe indicar quién es el proveedor de servicios y donde está su residencia legal, así como el enlace a su política de privacidad.

Los cuestionarios se recogen a través de la plataforma Qualtrics (https://www.qualtrics.com/es/nucleo-de-investigacion/encuestas-en-linea/). La cual, garantizada la seguridad de los datos mediante la codificación de estos, redundancia, supervisión continua de la red e inicio de sesión único (SSO). Así mismo, cuenta con la certificación ISO 27.001 y la autorización del programa FedRAMP. En los siguientes enlaces se detallan los aspectos relacionados con la seguridad y la protección de datos:

- https://www.qualtrics.com/platform/security/
- https://www.qualtrics.com/support/es/survey-platform/getting-started/data-protection-privacy/

3.10 Medidas de seguridad de la información: Dispositivos

- Indicar si se va a utilizar algún tipo de dispositivo extraíble (USB portátil, disco duro externo, etc.) y si se van a encriptar

La información se almacenará en un disco duro externo y se encriptará a través de la aplicación Veracrypt.

- En el caso de que no se utilicen sistemas informáticos corporativos, indicar si se hacen copias de seguridad.

- Indique las medidas de seguridad para documentos en formato papel (custodia, acceso).

Los documentos en formato papel recogidos en el estudio se custodiarán bajo llave en el despacho 26 de la Facultad de Ciencias Sociales y Humanas de la Universidad de Zaragoza (referencia de localización 004129). Una vez finalizado la investigación y cumplido el plazo de conservación establecido, los documentos serán destruidos mediante una trituradora de papel o mediante un servicio autorizado de destrucción de documentación confidencial.

Recomendaciones generales sobre uso de datos

- No utilizar redes Wifi para transmitir información sensible.
- Utilizar contraseñas fuertes y cambiarlas periódicamente.
- Encriptar siempre la información sensible que vaya a enviarse por correo electrónico.
- Procurar que las versiones de los sistemas operativos y los aplicativos estén siempre actualizados.
- En los ordenadores personales, utilizar siempre antivirus y que este actualizado.
- No abrir nunca ficheros adjuntos a correos electrónicos en los que no identifiquemos al remitente.
- No utilizar redes sociales para comunicar información sensible.
- El teléfono móvil es un dispositivo poco seguro para el manejo de información sensible, y los antivirus que se pueden instalar ofrecen poca protección.
- Utilizar siempre que se pueda aplicaciones corporativas
- El uso de USB u otros dispositivos extraíbles está altamente desaconsejado

4. DESCRIPCIÓN DEL PROYECTO DE INVESTIGACIÓN (completar los campos o adjuntar protocolo completo con la información equivalente)

4.1 Tareas del equipo investigador

Explicar brevemente quién participa en el estudio, en calidad de qué y qué tareas va a realizar, así como su filiación (puesto de trabajo actual). Se debe presentar el cv y la firma de todos ellos en el anexo I

Si el estudio es multicéntrico debe presentarse un Anexo I por cada centro

Este estudio será llevado a cabo por un equipo investigador conformado por cuatro miembros:

- Osma López, Jorge J., Profesor titular de universidad, Universidad de Zaragoza. Como investigador principal, será responsable de la coordinación general del estudio, asegurando el correcto desarrollo de todas sus fases y la adecuada ejecución de las tareas asignadas. Supervisará clínicamente al equipo y el avance del estudio, garantizando el cumplimiento de los protocolos establecidos. En la fase inicial, dirigirá y colaborará en la revisión de la literatura científica y en la elaboración del protocolo de evaluación, asegurando la solidez metodológica del proyecto. Asimismo, se encargará de la supervisión de la redacción y envío de la documentación necesaria para la aprobación del estudio por parte de los Comités de Ética, así como del registro del Ensayo Controlado Aleatorizado (ECA) en clinicaltrials.
  Durante el proceso de intervención, se encargará de la supervisión y formación del terapeuta y personal en formación, asegurando la correcta aplicación de la intervención psicológica. Además, coordinará la corrección y codificación de datos en la base del estudio. Posteriormente, supervisará el análisis de datos para extraer conclusiones relevantes. Finalmente, supervisará, coordinará y gestionará la elaboración de artículos científicos y la presentación de resultados en congresos.

- Martínez Borba, Verónica., Profesora ayudante doctora, Universidad de Zaragoza. Como investigadora del equipo, desempeñará diversos papeles en la evaluación, supervisión y análisis de los datos del estudio. En la fase inicial, dirigirá y colaborará en la revisión de la literatura científica. Será responsable de la evaluación y selección de los sujetos, asegurando que los criterios de inclusión y exclusión se apliquen correctamente. Además, supervisará de la recogida de datos, coordinando la correcta implementación de los protocolos de evaluación establecidos. Asimismo, se encargará de la redacción y envío de la documentación necesaria para la aprobación del estudio por parte de los Comités de Ética. Durante la intervención, formará al terapeuta en la correcta aplicación de la intervención psicológica. Su labor se extenderá a la gestión, análisis y depuración de los datos. Respecto a la difusión de resultados desempeñará un papel esencial en la redacción de artículos científicos y en la presentación de los resultados en congresos.
- Garcés Arilla, Sara., Personal investigador de la Universidad de Zaragoza. Gestionará los datos y la recogida de muestras biológicas dentro del estudio. Será responsable de la recogida de muestras de cabello para el análisis de cortisol, asegurando la correcta manipulación y almacenamiento de estas. Así mismo, administrará las pruebas para evaluar el estado cognitivo de participantes. Participará en la organización, codificación y gestión de los datos en la base de datos del estudio, garantizando la integridad y calidad de la información recopilada. Apoyará en el análisis estadístico, colaborando en la estructuración de los datos para su correcta interpretación. También contribuirá en la corrección y depuración de la base de datos. Finalmente, participará en la elaboración de informes y artículos científicos, colaborando en la difusión de los resultados obtenidos.
- Rodríguez Márquez, Andrés E., Personal investigador de la Universidad de Zaragoza. Cumplirá la función de apoyar en la evaluación y selección de participantes para el proyecto. Apoyará el registro y gestión de la base de datos, realizará las evaluaciones iniciales y aplicará la intervención grupal basada en el Protocolo Unificado. Posteriormente, colaborará en el análisis de los datos obtenidos, apoyando la interpretación de los resultados y su aplicación en el estudio. Además, participará en la elaboración y redacción de artículos científicos. Así como, apoyará la presentación de resultados en congresos.
- Del Corral Beamonte, Esther., Licenciada en Medicina y cirugía general, Hospital Royo Villanova. Durante los últimos 5 años desarrolla su labor en la unidad de aislamiento de alto nivel, en la consulta de enfermedades infecciosas y en la consulta de CPCOVID. Ha dirigido 4 proyectos centrado en CPCOVID o COVID-19. Su participación en el proyecto es fundamental para el reclutamiento de las y los participantes que visita en su centro.

4.2 Justificación del estudio: Antecedentes, estado actual del tema, relevancia (Citar las referencias bibliográficas en el apartado siguiente)

La enfermedad COVID-19 ha provocado una de las mayores pandemias de la historia mundial, con consecuencias sanitarias, sociales y económicas de gran impacto. Dos años y medio después de que la Organización Mundial de la Salud (OMS) declarara la pandemia, se ha constatado que un 10-20% de los pacientes con COVID-19 no logra recuperar su estado de salud previo y desarrolla una sintomatología persistente en el tiempo. Esta condición ha sido definida por la OMS como condición post-COVID-19 (CPCOVID) o COVID-19 Persistente, caracterizada por la presencia de síntomas físicos, cognitivos y emocionales que pueden mantenerse durante meses e incluso años después de la infección aguda. Entre los síntomas más prevalentes se encuentran la fatiga, dificultad para respirar y disfunción cognitiva, además de secuelas psicológicas significativas. Dentro de estas, los trastornos emocionales (TEs) son los más comúnmente reportados en esta población, incluyendo trastornos de ansiedad, trastornos depresivos y relacionados. Se estima que entre el 10-20% de las personas con COVID-19 desarrollan CPCOVID (OMS, 2023). En Aragón, las estimaciones son del 10% de las personas

diagnosticadas con COVID-19, lo que supone que un total de unas 46000 personas aproximadamente padecen CPCOVID (Gobierno de Aragón, 2022).

Siguiendo la literatura, las personas afectadas con CPCOVID presentan factores asociados a una experiencia de estrés crónico: incertidumbre sobre el curso de la enfermedad y la adaptación a nuevas condiciones de salud y la preocupación por las consecuencias a largo plazo (Szabo, 2023). En relación con este dato, se han encontrado niveles significativamente elevados de cortisol en pelo en personas con diferentes enfermedades crónicas (p. ej., dolor crónico [Jacobsen et al., 2023] y esclerosis múltiple [Pereira et al., 2019]) y se ha demostrado que estos niveles elevados influyen significativamente en la calidad de vida de los afectados (Wosu et al., 2013). Por otro lado, niveles elevados de cortisol se han relacionado con una peor función mnésica en condiciones médicas diversas (Assayag et al., 2017; Saleem et al., 2017). A este respecto, investigadoras que forman parte del equipo del presente proyecto han demostrado que las personas afectadas con CPCOVID presentan quejas subjetivas de memoria y que estas quejas, se relacionan de forma significativa con sus síntomas emocionales (Llana et al., 2023). A su vez, se han descrito alteraciones en el hipocampo tras la infección que ocasiona el CPCOVID que se relacionan con las alteraciones en la memoria y la sintomatología ansiosa y depresiva (Zorzo et al., 2023; para una revisión). Esta región cerebral se ve particularmente afectada por niveles elevados de cortisol (Jo.ls et al., 2008). Pese a que existen datos que apuntan a que la respuesta emocional y de cortisol, así como los déficits mnésicos, podrían ser factores que interactúan en la definición de los síntomas clásicamente asociados al CPCOVID, hasta el momento, no se han estudiado los niveles de cortisol en respuesta al estrés crónico en personas con esta condición.

Además de las alteraciones físicas y cognitivas descritas anteriormente, la pandemia en sí y las medidas de restricción tomadas, como el confinamiento o reducción del contacto social, así como la inestabilidad laboral y dificultades económicas experimentadas, tuvieron un impacto en el bienestar psicológico de la población general (Liu et al., 2021). Así, un estudio de metaanálisis con muestra de nueve países diferentes (n = 146139) informó de que los síntomas más comunes durante la pandemia por COVID-19 fueron los Trastornos Emocionales (TEs; nomenclatura que agrupa trastornos de ansiedad, depresión y relacionados; Bullis et al., 2019) (Liu et al., 2021).

En el caso particular de las personas que padecen CPCOVID, los problemas de salud mental notificados con mayor frecuencia fueron también los trastornos de ansiedad (22,2%) y depresión (21,1%); (Deer et al., 2021). A su vez, las personas que padecen CPCOVID podrían tener un riesgo de cronificación de su problema emocional, debido a tener que hacer frente no sólo a los síntomas físicos de la enfermedad, sino también a la soledad y al estigma relacionado con el escepticismo sobre sus síntomas, lo que a su vez podría afectar a su vida social y familiar, su funcionamiento laboral y su calidad de vida (Brown y O'Brien, 2021; Office of National Statistics, 2021). Así, se observa en personas con CPCOVID una reducción de las actividades de la vida diaria y una disminución de la calidad de vida (de Oliveira Almeida et al., 2022). Por todo lo expuesto, se hace necesario el abordaje integral de estas personas, siendo los tratamientos psicológicos de gran ayuda para el manejo del malestar emocional, la recuperación funcional y la mejora en la calidad de vida.

En esta línea, la implementación de intervenciones transdiagnósticas como el Protocolo Unificado para el Tratamiento Transdiagnóstico de los TEs (PU; Barlow et al., 2011, 2018), podría servir para el mencionado propósito. Este protocolo se basa en los principios de la Terapia Cognitivo-Conductual (TCC) y el objetivo principal de este protocolo es disminuir el malestar en respuesta a las emociones intensas y reducir las reacciones desadaptativas ante las emociones de cualquier tipo de valencia. Por tales motivos, el PU se focaliza en mostrar el valor adaptativo de todas las emociones, en identificar y modificar las estrategias desadaptativas utilizadas por la persona para evitar o disminuir la intensidad de las emociones y aumentar la tolerancia a las

emociones intensas. Este protocolo destaca que las consecuencias de las estrategias de evitación emocional a medio y largo plazo son muy negativas para la persona, no solo porque mantienen el problema, sino que también generan interferencias en el día a día de la persona, generando una atmósfera aún más problemática. El PU consta de ocho módulos diseñados para entrenar cinco habilidades fundamentales de regulación emocional: conciencia plena, flexibilidad cognitiva, oposición a las conductas emocionales desadaptativas, exposición interoceptiva y exposición emocional (Barlow et al., 2018). Su estructura modular permite una mayor flexibilidad y adaptabilidad a diversos problemas clínicos, facilitando su aplicación en diferentes contextos terapéuticos (p. ej., Martínez-Borba et al., 2022; Sauer-Zavala et al., 2021). En cuanto a su utilidad clínica en la mejora de los TEs en la población general, diversas revisiones sistemáticas y meta-análisis han respaldado su efectividad (Sakiris & Berle, 2019; Cassiello-Robbins & Carlucci et al., 2021; Longley & Gleiser, 2023). En España, su aplicación en formato grupal dentro de unidades de salud mental públicas ha evidenciado su eficacia y coste-efectividad en el tratamiento de los TEs, consolidándose como una opción terapéutica eficiente (Peris-Baquero et al., 2022; Peris-Baquero & Osma, 2023). Además, su implementación en formatos costo-efectivos, como la terapia grupal y las intervenciones en línea, ha demostrado ser viable y eficaz (p. ej., Reinholt et al., 2017; Schaeuffele et al., 2022).

Por otro lado, distintos estudios apuntan que niveles elevados de disregulación emocional se asocian directamente con diferentes trastornos médicos (Smith & MacKenzie, 2006; Suls et al., 2005). Así, un estudio de revisión sistemática informó de que el PU podría ser eficaz para tratar la sintomatología emocional en una población que presenta, a su vez, un trastorno médico, implicando también mejoras en la sintomatología médica en algunos estudios (Osma et al., 2021). A este respecto, la regulación emocional ha sido sugerida como un posible mecanismo transdiagnóstico para la salud mental en el contexto de COVID-19 (Volkert et al., 2021). Considerando estos datos, se puede hipotetizar que el PU podría resultar útil para la mejora de la sintomatología emocional y física en personas con CPCOVID.

4.3 Bibliografía (debe estar referenciada en el texto anterior)

Barlow, D. H., Farchione, T. J., Fairholme, C. P., Ellard, K. K., Boisseau, C. L., Allen, L. B., & Ehrenreich-May, J. T. (2011). Unified protocol for transdiagnostic treatment of emotional disorders: Therapist guide. Oxford University Press.

Barlow, D. H., Farchione, T. J., Sauer-Zavala, S., Latin, H. M., Ellard, K. K., Bullis, J. R., Bentley, K., Boettcher, H., y Cassiello-Robbins, C. (2018). Unified protocol for transdiagnostic treatment of emotional disorders: Therapist guide (2nd ed.). New York, NY: Oxford University Press.

Bullis, J. R., Boettcher, H., Sauer-Zavala, S., Farchione, T. J., & Barlow, D. H. (2019). What is an emotional disorder? A transdiagnostic mechanistic definition with implications for assessment, treatment, and prevention. Clinical Psychology: Science and Practice, 26(2), 1–19. https://doi.org/10.1111/cpsp.12278

Cassiello-Robbins, C., Southward, M. W., Tirpak, J. W., y Sauer-Zavala, S. (2020). A systematic review of Unified Protocol applications with adult populations: Facilitating widespread dissemination via adaptability. Clinical Psychology Review, 78, 101852. https://doi.org/10.1016/j.cpr.2020.101852

Sakiris, N., y Berle, D. (2019). A systematic review and meta-analysis of the Unified Protocol as a transdiagnostic emotion regulation based intervention. Clinical psychology review, 72, 101751. https://doi.org/10.1016/j.cpr.2019.101751

Deer, R. R., Rock, M. A., Vasilevsky, N., Carmody, L., Rando, H., Anzalone, A. J., Basson, M. D., Bennett, T. D., Bergquist, T., Boudreau, E. A., Bramante, C. T., Byrd, J. B., Callahan, T. J., Chan, L. E., Chu, H., Chute, C. G., Coleman, B. D., Davis, H. E., Gagnier, J., Greene, C. S., … Robinson, P. N. (2021). Characterizing Long COVID: Deep Phenotype of a Complex Condition. EBioMedicine, 74, 103722. https://doi.org/10.1016/j.ebiom.2021.103722

Joëls, M., Karst, H., DeRijk, R., & de Kloet, E. R. (2008). The coming out of the brain mineralocorticoid receptor. Trends in Neurosciences, 31(1), 1–7. https://doi.org/10.1016/j.tins.2007.10.005

Llana, T., Mendez, M., Garces-Arilla, S., Hidalgo, V., Mendez-Lopez, M. (2023). Association between olfactory dysfunction and mood disturbances with objective and subjective cognitive deficits in long-COVID. Frontiers in Psychology, 14, 1076743. https://doi.org/10.3389/fpsyg.2023.1076743

Longley, S. L., & Gleiser, T. S. (2023). Efficacy of the Unified Protocol: A systematic review and meta-analysis of randomized controlled trials. Clinical Psychology: Science and Practice, 30(2), 208.

Liu, X., Zhu, M., Zhang, R., Zhang, J., Zhang, C., Liu, P., et al. (2021). Public mental health problems during COVID-19 pandemic: a large-scale meta-analysis of the evidence. *Transl. Psychiatry* 11, 384–310. doi: 10.1038/s41398-021-01501-9

Martínez-Borba, V., Martínez-García, L., Peris-Baquero, Ó., Osma, J., y del Corral-Beamonte, E. (2023). Unified Protocol for the Transdiagnostic Treatment of Emotional Disorders in people with Post COVID-19 condition study protocol for a multiple baseline n-of-1 trial. Frontiers in Psychology, 14, 1160692.

Martínez-Borba, V., Martínez-García, L., Peris-Baquero, Ó., Osma, J., & del Corral-Beamonte, E. (2024). Guiding future research on psychological interventions in people with COVID-19 and post COVID syndrome and comorbid emotional disorders based on a systematic review. Frontiers in Public Health, 11, 1305463.

Martínez-Borba, V., Peris-Baquero, Ó., Martínez-García, L.Osma, J., y del Corral-Beamonte, E. (2024). Unified Protocol Application in Patients With Long COVID-19 Condition en Osma y Farchione (Ed.), Applications of the Unified Protocol in Health Conditions. Oxford University Press. https://global.oup.com/academic/product/applications-of-the-unified-protocol-inhealth-conditions-9780197564295?cc=pt&lang=en&#

Osma, J., Martínez-García, L., Quilez-Orden, A., & Peris-Baquero, Ó., (2021). Unified protocol for the transdiagnostic treatment of emotional disorders in medical conditions: A systematic review. International Journal of Environmental Research and Public Health, 18(10), 5077.

Peris-Baquero, Ó., y Osma, J. (2023). Unified Protocol for the Transdiagnostic Treatment of Emotional Disorders in Group Format in Spain: Results of a Noninferiority Randomized Controlled Trial at 15 Months after Treatment Onset. Depression and Anxiety, 2023.

Schaeuffele, C., Homeyer, S., Perea, L., Scharf, L., Schulz, A., Knaevelsrud, C., ... & Boettcher, J. (2022). The unified protocol as an internet-based intervention for emotional disorders: Randomized controlled trial. PloS One, 17(7), e0270178.

Schaeuffele, C., Meine, L. E., Schulz, A., Weber, M. C., Moser, A., Paersch, C., ... & Kleim, B. (2024). A systematic review and meta-analysis of transdiagnostic cognitive behavioural therapies for emotional disorders. Nature human behaviour, 1-17.

Szabo, S. (2023). The post–COVID stress syndrome: From the three-stage stress response of Hans Selye to COVID-19. Inflammopharmacology, 31, 2799–2806. https://doi.org/10.1007/s10787-023-01179-z

Volkert, J., Taubner, S., Berning, A., Kling, L., Wießner, H., Georg, A. K., et al. (2021). Transdiagnostic mechanisms of mental health during the COVID-19 pandemic on adults and families in Germany: study protocol of a cross-sectional and 1-year longitudinal study. Front. Psychol. 12, 1–11. https://doi.org/10.3389/fpsyg.2021.720104

Zorzo, C., Solares, L., Mendez, M., Mendez-Lopez, M. (2023). Hippocampal alterations after SARS-CoV-2 infection: A systematic review. Behavioural Brain Research, 455,114662. https://doi.org/10.1016/j.bbr.2023.114662

4.4 Hipótesis (afirmación que se pretende demostrar)

Hipótesis:

- *H1 (OE1):* Las personas participantes en el grupo experimental (Programa psicológico basado en el Protocolo Unificado) mostrarán una reducción significativa de los síntomas emocionales (ansiedad, depresión, estrés, disregulación emocional, dimensiones de los trastornos emocionales, quejas de memoria) y una mejora significativa en calidad de vida, en comparación con el grupo de control.
- *H2 (OE2)*: Las mejorías clínicas obtenidas en la evaluación post-programa se mantendrán en los seguimientos a los 3, 6 y 12 meses tras la intervención.
- *H3 (OE3):* Se encontrarán altas tasas de satisfacción y una alta adherencia a la intervención psicológica basada en el Protocolo Unificado.
- *H4 (OE4):* Los resultados del análisis longitudinal de los niveles de cortisol y cortisona (estrés a nivel endocrino) proporcionará conocimientos cruciales sobre cómo la terapia psicológica basada en el Protocolo Unificado puede contribuir al bienestar de las personas que padecen CPCOVID.

4.5 Objetivos

Objetivo General:

Evaluar la eficacia y aceptabilidad del Protocolo Unificado (PU) en formato grupal online para el tratamiento de los trastornos emocionales en 90 personas adultas con condición CPCOVID y con diagnóstico de trastorno emocional, atendidos en el Servicio Aragonés de Salud.

Objetivos Específicos:

- *OE1*: Llevar a cabo un ensayo clínico aleatorio (ECA) para comparar la eficacia del Protocolo Unificado en formato grupal online para mejorar la sintomatología emocional (ansiedad, depresión, estrés, disregulación emocional, dimensiones de los trastornos emocionales), la calidad de vida y las quejas de memoria en personas con CPCOVID frente a un grupo control.
- *OE2:* Explorar la estabilidad de los cambios obtenidos tras la intervención a lo largo del tiempo, evaluando su impacto en 3, 6 y 12 meses después del tratamiento.

- *OE3:* Analizar la aceptabilidad y satisfacción de los participantes tras recibir el Protocolo Unificado en formato grupal online.
- *OE4:* Evaluar la evolución en los niveles de estrés crónico a través de los niveles de cortisol y cortisona acumulados en pelo antes y después de la aplicación del Protocolo Unificado en formato grupal.

4.6 Metodología (se deben detallar **todos** los campos siguientes**):**
Diseño del estudio
Participantes: criterios de inclusión/exclusión; modo de reclutamiento (quién y cómo realiza el contacto inicial con los participantes, presentar material de difusión del estudio, si lo hay), tamaño muestral (y su justificación), aleatorización (si procede)
Fuentes de información: variables detalladas (datos a recoger), origen de los datos, cuándo y cómo se recogen, a qué periodo de tiempo se refieren.
Procedimientos: detallar de forma diferenciada los procedimientos puramente asistenciales de los propios de la investigación, presentar encuestas o formularios que se vayan a utilizar (link en caso de ser encuestas online), valoración del riesgo de los procedimientos experimentales y medidas para minimizarlo.
Análisis estadístico
Consideración de la perspectiva de género: detallar las medidas adoptadas para que los resultados del estudio puedan reflejar posibles diferencias por sexo/género.
Limitaciones del estudio
En caso de muestras biológicas: detallar tipo y número de muestras, cómo se recogen, dónde y quién las analiza, cuándo se destruyen (o destino final)

Diseño:
Se llevará a cabo un Ensayo clínico aleatorizado (ECA) paralelo con dos condiciones 1:1; con un grupo experimental (grupo de tratamiento inmediato) que recibe una intervención psicológica basada en el Protocolo Unificado y grupo control lista de espera (con tratamiento demorado).

Participantes:
- *Criterios de inclusión:*
  - Pertenecer a la comunidad autónoma de Aragón.
  - Tener al menos 18 años.
  - Comprender bien el castellano.
  - Infección por SARS-CoV-2 documentada mediante PCR, test de antígenos o serología (Ac anti-N positivos).
  - Persistencia de los síntomas físicos más allá de 12 semanas tras la infección aguda por SARS-CoV-2.
  - Presentar una puntuación de 8 o más en la escala OASIS de ansiedad y/o una puntuación de 7 o más en la escala ODSIS de depresión.
  - Cumplir criterios diagnósticos de trastorno emocional.
  - Disponer de acceso a Internet.
  - Firma del consentimiento informado.
- *Criterios de exclusión:*
  - Síntomas emocionales preexistentes a la infección aguda por SARS-CoV-2.
  - Estar recibiendo tratamiento psicológico actualmente.
  - Tener un diagnóstico de trastorno mental grave (p. ej., trastorno de personalidad, trastorno bipolar, etc.).
  - Presentar ideación suicida activa en el momento de la evaluación.

- Las personas en tratamiento con psicofármacos deberán mantener la dosis durante el tiempo que dure el estudio, salvo contraindicación médica.

- *Tamaño muestral:* El estudio contará con un tamaño muestral total de 90 personas participantes, distribuidas equitativamente en dos grupos:
  - 45 personas recibirán la intervención basada en el Protocolo Unificado en formato grupal online. Además, dentro de este grupo, se invitará a las/os participantes a proporcionar una muestra de cabello, antes y después de recibir el programa psicológico basado en el Protocolo Unificado. Estas muestras permiten medir los niveles de cortisol y cortisona, unas hormonas vinculadas con el estrés para así analizar posibles cambios a lo largo de la intervención. En la sesión presencial de recogida de cabello también se administrará a las/os participantes medidas de funcionamiento cognitivo.
  - Las 45 personas restantes, no recibirán la intervención psicológica inmediata, sino que permanecerán en una lista de espera durante 12 semanas aproximadamente. Al finalizar este periodo, serán evaluadas nuevamente y pasarán a recibir la intervención basada en el Protocolo Unificado en formato grupal online.

Utilizando el programa informático G*Power (Faul et al., 2007), y teniendo en cuenta las dos condiciones, los 5 momentos de evaluación (pretratamiento, post-tratamiento, y seguimientos a los 3, 6 y 12 meses) y los modelos estadísticos que se aplicarán para analizar los datos, hemos obtenido un tamaño de muestra total de 74 participantes con una potencia estadística del 90% y un coeficiente alfa de 0,05 y un tamaño del efecto de 0,30. Considerando una tasa de abandono del 20%, se estima un tamaño de muestra de 45 participantes por condición (N total = 90).

- *Aleatorización:* La asignación de las personas participantes a las condiciones experimentales y de control se llevará a cabo mediante procedimientos de aleatorización utilizando el software Randomizer.

Fuentes de información:

Pruebas administradas para determinar los criterios de inclusión (participantes del grupo experimental y control):

- **Entrevista estructurada para los trastornos de ansiedad y trastornos relacionados, según el DSM-5 (ADIS-5; Brown y Barlow, 2014):** Entrevista estructurada que evalúa los criterios DSM-5 para los trastornos de ansiedad, estado de ánimo y relacionados.

Pruebas administradas online a través de la plataforma qualtrics (participantes del grupo experimental y control):

- **Cuestionario sociodemográfico y de historia clínica (ad hoc):** género, edad, lugar de residencia (código postal), estado civil, situación laboral (profesión), estatus socioeconómico, peso, estatura, estatus menstrual, hábitos de sueño, hábitos de vida (tabaco, alcohol, actividad física, dieta), vacunación SARS CoV 2 (Si/No previo a infección, número de dosis y fecha de estas, marca de la vacuna).
- **Long COVID Pre Assessment Questionnaire (National Health Service, 2021):** cuestionario en el que se evalúan distintos síntomas físicos presentes en la CPCOVID.
- **Escala General de Gravedad e interferencia para la Depresión (ODSIS; Osma et al., 2019):** frecuencia,

intensidad, gravedad e interferencia de la sintomatología depresiva.

- **Escala General de Gravedad e Interferencia para la Ansiedad (OASIS; Osma et al., 2019):** frecuencia, intensidad, gravedad e interferencia de la sintomatología ansiosa.
- **Inventario Multidimensional para los Trastornos Emocionales (MEDI; Osma et al., 2021):** perfil transdiagnóstico de los Trastornos Emocionales, compuesto por nueve dimensiones: temperamento neurótico, temperamento positivo, estado de ánimo deprimido, ansiedad somática, arousal, ansiedad social, cogniciones intrusivas, reexperimentación traumática, y evitación
- **Escala de Dificultades en la Regulación emocional (DERS; Hervás y Jodar, 2008):** dificultades en regulación emocional por medio de 5 subescalas (descontrol, rechazo, interferencia, desatención y confusión emocional).
- **Escala de tolerancia al estrés (DTS; Sandín et al., 2017):** evaluación a través de 15 ítems de la tolerancia al malestar. Evalúa las siguientes dimensiones: 1) Capacidad percibida para tolerar el malestar emocional; 2) Valoración subjetiva del malestar; 3) Atención absorbida por las emociones negativas; 4) Esfuerzos de regulación para aliviar el malestar.
- **Cuestionario fallos de memoria de la vida diaria (MFE; Montejo et al., 2012):** evaluación de los olvidos en la vida cotidiana.
- **EuroQol-5D (Badia et al., 1999):** estado de salud auto-percibido.
- **Escala de Percepción del Estrés (PSS; Remor y Carrobles, 2001):** percepción del estrés experimentado en el último mes.
- **Cuestionario de Satisfacción con el Tratamiento (STQ; ad hoc):** satisfacción con el programa recibido, en base a su calidad percibida, adecuación a sus expectativas, su recomendación del programa a seres queridos, la utilidad de las técnicas aprendidas, y su satisfacción general con el programa y su formato. Se administra únicamente tras la administración del programa (T2).
- **Cuestionario de evaluación de los módulos del PU (ad hoc):** pregunta de carácter general que evalúa la utilidad del programa para mejorar la regulación emocional y seis preguntas específicas que evalúan por separado la utilidad para regular mejor las emociones de cada una de las técnicas que se trabajan en los diferentes módulos del PU. La escala de respuesta es tipo Likert y va de 0 (nada) a 10 (muchísimo). Se administra únicamente tras la administración del programa (T2).

Pruebas administradas presencialmente (participantes del grupo experimental):

- **Montreal Cognitive Assessment (MoCA; Nasreddine et al., 2005):** prueba neuropsicológica breve diseñada para evaluar el deterioro cognitivo leve. Evalúa múltiples dominios cognitivos, incluyendo habilidades visuoespaciales, memoria, memoria de trabajo, atención, concentración, lenguaje, funciones ejecutivas y orientación.
- **Test de dígitos y símbolos: subtest de la Wechsler Adult Intelligence Scale-III (WAIS-III; Wechsler, 1997):** el subtest de dígitos analiza la capacidad de atención, la memoria a corto plazo y la memoria de trabajo, mediante la repetición de secuencias numéricas en orden directo e inverso, lo cual permite observar la habilidad para retener y manipular información auditiva de forma inmediata. Por su parte, el subtest de símbolos (Claves) evalúa la velocidad de procesamiento, la atención sostenida, la coordinación visomotora y el aprendizaje asociativo inmediato, pidiendo a la persona que relacione números con símbolos siguiendo una clave y en un tiempo limitado.
- **Subtest de dígitos; subtest de la Wechsler Adult Intelligence Scale-IV (WAIS-IV; Wechsler, 2008):** la tarea de dígitos en orden directo de este subtest evalúa la memoria a corto plazo verbal y consiste en una tarea de recuerdo numérico que mide la repetición mecánica de una secuencia de números.
- Cuestionario **de recogida de información relevante sobre características y tratamientos capilares de las personas participantes (ad hoc):** este instrumento incluye preguntas relativas al estado actual del

cabello, tales como si está tratado, si han recibido tratamientos químicos, y si presentan problemas de caída capilar, entre otros aspectos.

Procedimientos:

El reclutamiento de las personas participantes se realizará a través del Hospital Royo Villanova de Zaragoza. La Dra. Esther del Corral Beamonte ofrecerá a las y los pacientes atendidos presencialmente en su consulta la posibilidad de participar en el estudio. Los criterios de inclusión que tendrá en cuenta la Dra. Esther del Corral Beamonte son que las/os posibles participantes: residan en la Comunidad Autónoma de Aragón, sean mayores de edad, comprendan el castellano, tengan infección por SARS-CoV-2 documentada y diagnóstico de COVID-19 persistente, dispongan de acceso a Internet y estén dispuestos a recibir el programa psicológico en formato grupal online. Las personas que cumplan estos criterios de inclusión iniciales recibirán un díptico informativo para acceder a la plataforma online Qualtrics. Aquellas/os participantes que tengan interés en participar en el estudio tendrán que entrar en el enlace o código QR que se muestra en el díptico para acceder a la plataforma online Qualtrics donde encontrarán el documento de información y el consentimiento informado (se encuentra adjunto). Tras la firma del consentimiento informado online, le aparecerá a cada participante un código numérico aleatorio. Una vez firmado el consentimiento, el psicólogo encargado de llevar a cabo el programa psicológico (Andrés Rodríguez Márquez) contactará con las participantes por correo y teléfono para concertar una sesión online a través de videollamada. Esta sesión online e individual tendrá una duración aproximada de 60 a 90 minutos, y servirá para realizar la entrevista clínica donde se confirmarán los criterios de inclusión y exclusión para participar en el estudio (puntuación en ODSIS y OASIS, cumplir criterio de diagnóstico de trastorno emocional, no presentar un trastorno mental grave ni ideación suicida activa). A los y las participantes que sean excluidos/as del estudio se les informará de posibles alternativas de intervención más ajustadas a su problemática actual. Tras la entrevista, se enviará por email un enlace de Qualtrics para completar el protocolo de evaluación pre-intervención (T1). Posteriormente, se les informará por email de la condición a la que han sido asignados aleatoriamente (grupo experimental o grupo control lista de espera).
A las y los participantes del grupo experimental se les invitará a participar en la recogida de muestra de cabello, para determinar cambios en los niveles de cortisol y cortisona acumulados en tres momentos de medida (pre-tratamiento; tres meses tras el fin del tratamiento; y 12 meses tras el fin del tratamiento). Este procedimiento se realizará de forma individual y presencial con Sara Garcés Arilla. Así mismo, en esta sesión presencial las personas seleccionadas completarán el MoCA, el subtest del WAIS III (dígitos y símbolos), el subtest del WAIS IV (dígitos) y el cuestionario ad hoc sobre cuestiones capilares.

Tras responder a la evaluación pre-programa (y proporcionar la muestra de cabello en el caso del grupo experimental), las/os participantes recibirán la intervención psicológica basada en el Protocolo Unificado. Este procedimiento será diferente en función de la condición a la que la persona ha sido asignada:

- <u>Grupo experimental-Intervención psicológica basada en Protocolo Unificado (PU) en formato grupal online</u>: las personas asignadas a este grupo recibirán un breve manual escrito de apoyo con los contenidos a trabajar en la intervención psicológica y asistirán a 12 sesiones de la intervención psicológica basada en el PU en formato grupal online, a través de la plataforma Google Meet. Las sesiones se llevarán a cabo una vez por semana, con una duración de 2 horas cada una de ellas. La estructura de la intervención psicológica y contenido de las sesiones que recibirá es la siguiente:
    - Módulo 1 (1 sesión): establecimiento de objetivos y motivación para el cambio.
    - Módulo 2 (2 sesiones): comprendiendo tus emociones y análisis de las emociones.
    - Módulo 3 (2 sesiones): conciencia emocional.

- Módulo 4 (2 sesiones): flexibilidad cognitiva.
- Módulo 5 (1 sesión): oponiéndose a las conductas emocionales.
- Módulo 6 (1 sesión): exposiciones interoceptivas.
- Módulo 7 (2 sesión): exposiciones emocionales.
- Módulo 8 (1 sesión) prevención de recaídas.

Una vez finalizada la intervención psicológica se solicitará a las personas que accedan a un enlace de Qualtrics para rellenar la evaluación post-intervención (T2), con una duración aproximada de 25 a 30 minutos. Así mismo se llevarán a cabo tres sesiones de seguimiento, a los 3 (T3), 6 (T4) y 12 (T5) meses tras finalizar la intervención.

- Grupo control-Lista de espera: las personas asignadas a esta condición, no recibirá la intervención psicológica de forma inmediata. En cambio, tras la evaluación pre-programa, permanecerán en lista de espera durante 12 semanas. Una vez finalizado este periodo de espera, realizarán la evaluación post-programa a través de Qualtrics y recibirán la intervención psicológica basada en el PU en formato grupal online. La duración, contenidos de las sesiones y procedimientos serán exactamente iguales a los descritos en la condición del grupo experimental “Intervención psicológica basada en el PU en formato grupal online”.

Puesto que el programa que reciben las participantes del grupo experimental consiste en asistir de manera síncrona a un programa psicológico proporcionado en formato grupal no es posible realizar el cegamiento recomendado en los ECA. Así, tanto las/os participantes como el psicólogo encargado de aplicar el programa psicológico serán conocedores de la condición a la que han sido asignados/as los/as participantes. No obstante, para reducir la posibilidad de sesgos en la intervención, los/as participantes serán asignados aleatoriamente a cada una de las condiciones (evita el sesgo de elección) y parte de la evaluación se realizarán mediante una plataforma online y cuestionarios autorreportados (se evita el sesgo del evaluador/a).

Análisis estadísticos:

Estadísticos descriptivos, correlaciones r de Pearson, regresiones lineales múltiples, y modelos lineales de efectos mixtos mediante el paquete lm4 (versión lme4_1.1-13; Bates et al., 2015) para el software estadístico R (versión 4.1.0; R Core Team, 2021) y SPSS v25.0 (IBM Corp, 2017). Teniendo en cuenta la naturaleza de las variables que se van a incluir en los modelos, se emplearán varios modelos lineales de efectos mixtos para cada variable dependiente a analizar (cada uno de los distintos cuestionarios empleados en las mediciones pre y post intervención). Para cada uno de estos modelos, el Tiempo (variable intrasujeto; T1 <pre tratamiento> vs. T2 <postratamiento> vs. T3 <3 meses> vs. T4 <6 meses> vs. T5 <12 meses>) y el Grupo Experimental (variable intersujeto; Experimental vs. Lista de espera) se tendrán en cuenta como efectos fijos del modelo. Se tendrán también en cuenta pendientes aleatorias para los/las participantes, que serán incluidos como efectos aleatorios del modelo. Los modelos tendrán la siguiente estructura: [Variable Dependiente ~ Tiempo × Grupo Experimental + (1|Participante)].
Para las muestras de cabello se realizarán ANOVA de medidas repetidas con un diseño factorial. Se establecerá aproximación correlacional para determinar asociaciones entre el cortisol y otras variables dependientes de tipo emocional y las quejas de memoria.

Consideración perspectiva de género:

Cabe destacar que la incidencia del CPCOVID y de los TEs es mayor en el caso de las mujeres. En este sentido,

los resultados de un estudio llevado a cabo en España con 341 personas con CPCOVID (Mateu et al., 2023), encontraron que el 69.8% eran mujeres (prevalencias similares a las obtenidas en otros estudios como Nielsen et al., 2022), y que ser mujer, junto con tener antecedentes de cefalea, taquicardia, fatiga, quejas neurocognitivas o disnea predecían el desarrollo de CPCOVID, y una menor probabilidad de recuperarse del CPCOVID (Mateu et al., 2023). De una manera similar, la prevalencia de los TEs tiene una mayor incidencia sobre las mujeres, de hecho, el diagnóstico de trastornos depresivos es el doble de prevalente su diagnóstico en mujeres (Santomauro et al., 2021). Padecer CPCOVID y además un TE se ha asociado con deterioros importantes en la calidad de vida (Malick et al., 2021), disminuciones del estado funcional en el 94% de los casos (Nielsen et al., 2022) y más de un 56% de bajas laborales (Nielsen et al., 2022).

Este estudio se pretende abordar las desigualdades sociales y de género en salud con la inclusión de la variable género (junto con otras variables sociodemográficas y clínicas) en todos los análisis estadísticos que se realicen y, de esta manera, contribuir al avance en el conocimiento sobre las diferencias de género en el CPCOVID y en la psicopatología de los TEs y en la coste-eficacia, adherencia y aceptabilidad de las intervenciones psicológicas para estos trastornos. Para la elaboración de todos los trabajos científicos se tendrán en cuenta las variables género y edad de todas las personas participantes. Este conocimiento servirá para diseñar adaptaciones futuras de las intervenciones psicológicas en función del género (personalización de los tratamientos) y contribuir así a reducir las desigualdades de género en salud mental.

Limitaciones del estudio:

A lo largo de la puesta en marcha del estudio podrán surgir algunas limitaciones que deberán tenerse en cuenta en la interpretación de los resultados. En primer lugar, uno de los principales obstáculos identificados es el posible aumento de carga de trabajo para las/os profesionales colaboradores encargados de facilitar la muestra. Para minimizar esta sobrecarga, las evaluaciones se realizarán preferentemente en formato online mediante la plataforma Qualtrics, lo que permitirá el almacenamiento automático y seguro de los datos. En segundo lugar, podría producirse pérdida de información durante las evaluaciones. Sin embargo, el formato digital de recogida de datos está configurado para evitar omisiones, asegurando que todas las preguntas sean completadas antes de enviar los formularios. Otra limitación que considerar es la posibilidad de encontrar valores anómalos en los análisis de las muestras capilares, lo cual podría afectar a la interpretación de los niveles de cortisol. Para ello, se incorpora un cuestionario específico sobre tratamientos capilares y se medirán también los niveles de cortisona, con el fin de aumentar la validez interpretativa de los resultados. Por último, para garantizar la fidelidad a la hora de aplicar la intervención psicológica basada en el Protocolo Unificado, el terapeuta recibirá una sesión de formación complementaria, así como sesiones de supervisión continuada, orientadas a la resolución de dudas, la motivación y el seguimiento de la fidelidad en la aplicación del protocolo.

Muestras biológicas:

El procedimiento de toma de muestras biológicas consistirá en la toma de 8 a 10 cabellos de la parte posterior de la cabeza. Para obtener dicha muestra, se cortarán cuidadosamente los cabellos de unos de tres centímetros de longitud de la parte posterior de la cabeza, cortados lo más cerca posible del cuero cabelludo. La muestra será analizada por el laboratorio Kirschbaum de la Technische Universität Dresden, ubicado en Alemania, especializado y con sello de calidad (https://dresden-labservice.com/quality-control/) para determinar el nivel de cortisol y cortisona acumulado durante los últimos tres meses. Las muestras se almacenarán en sobres de aluminio, identificados mediante un código anónimo para identificar la muestra, dentro de una bolsa tipo zip. Este tipo de muestras no requiere refrigeración, por lo que, posteriormente, se guardarán en un armario del laboratorio de psicobiología del Campus de Teruel de la Universidad de Zaragoza (referencia de localización

004129), al que solo tendrá acceso el personal encargado de la investigación. Una vez finalizado el proyecto, se destruirán las muestras o cualquier material sobrante. Cabe destacar que el análisis no tiene fines diagnósticos de ninguna enfermedad física ni psicológica.

4.7 Aspectos éticos (balance riesgo/beneficio, **justificación en caso de solicitar exención del consentimiento** informado, implicaciones asistenciales, implicaciones para el participante o su familia, compensación a los participantes, póliza de seguro).

Todas las personas que cumplan los criterios de inclusión y participen en el ensayo firmarán el documento de Protección de Datos para que tengan noción de quién va a utilizar los resultados de esta investigación y con qué fin. Así mismo, serán informadas sobre qué consiste la intervención psicológica, al igual que su duración y las fases del estudio. Para ello se les proporcionará la hoja de información explicando el tratamiento, y sus respectivas fases, así como el documento correspondiente al Consentimiento Informado.
En cuanto a la confidencialidad, el tratamiento, la comunicación y la cesión de los datos de carácter personal de todos los sujetos participantes, se ajustará a lo dispuesto en la Declaración de Helsinki (Seúl, 2008), Ley 14/2007 de Investigación biomédica, Ley Orgánica 3/2018, de 5 de diciembre, de Protección de Datos Personales y de Garantía de Derechos Digitales (LOPD GDD). A partir del 25 de mayo de 2018 es de plena aplicación la nueva legislación en la UE sobre datos personales, en concreto el Reglamento (UE) 2016/679 del Parlamento europeo y del Consejo de 27 de abril de 2016 de Protección de Datos (RGPD). De acuerdo con lo que establece la legislación mencionada, las personas participantes pueden ejercer los derechos de acceso, modificación, oposición y cancelación de datos, para lo cual deberá dirigirse al investigador principal a cargo del estudio. La información personal recogida para el estudio será sustituida por códigos numéricos y los datos sociodemográficos se guardarán de manera separada y solo se tendrá acceso por parte de los investigadores responsables del almacenamiento y tratamiento de los datos, siempre protegiendo el derecho a la privacidad.

La información de las medidas psicosociales y cognitivas se recogerá mediante la plataforma Qualtrics. Esta plataforma en concreto cumple con el RGPD y la CCPA (Ley de Privacidad del Consumidor de California). Qualtrics se compromete a mantener seguros los datos de los clientes y a brindar capacidades para ayudarlos a cumplir con cualquier regulación de privacidad y seguridad de datos a la que puedan estar sujetos.

En proyectos de investigación donde se aplica una intervención psicológica a menudo se incluye un grupo control que no recibe la intervención psicológica de manera demorada, sino que la recibe pasadas unas semanas desde que el grupo experimental empieza el programa. Esto permite determinar qué mejoras de las observadas en el grupo experimental se deben realmente al programa psicológico y cuáles se podrían explicar por otros factores como el paso del tiempo. Por cuestiones ética, y con el objetivo de que todos/as los participantes se puedan beneficiar del programa psicológico, se propone que el grupo control permanezca en lista de espera durante las 12 semanas que dura el programa psicológico, pudiendo realizar la intervención pasado este tiempo. Por este motivo, las medidas fisiológicas para analizar los niveles de cortisol en pelo no pueden ser recogidas en el grupo control. Los análisis de cortisol en pelo aportan un indicador de los niveles de cortisol durante los últimos 3 meses. Por este motivo, pasa conocer los niveles de cortisol en el momento post-programa hay que esperar tres meses (esta medida se recoge en el seguimiento a los tres meses y no el momento post-programa). Con el objetivo de que el grupo control se pueda beneficiar cuanto antes del programa, se ha optado por no recoger las medidas de cortisol en este grupo. Al mismo tiempo, con el objetivo de reducir

la carga de realizar evaluaciones psicológicas extensas, especialmente cuando los/as participantes no se benefician del programa psicológico, los participantes del grupo control no realizan la evaluación presencial donde se administran las medidas de inteligencia y deterioro cognitivo.

4.8 Cronograma y plan de trabajo:

- Etapas de desarrollo, duración, fechas estimadas de inicio y fin (indicar al menos mes y año).
- Lugares donde se prevé realizar el proyecto, instalaciones que se utilizarán.

	**2025**				**2026**			
**Tareas**	**1**	**2**	**3**	**4**	**1**	**2**	**3**	**4**
Preparación documentación y solicitud de aprobación de la realización del estudio por parte del Comité Ético de Investigación Clínica de Aragón	■							
Registro del ECA en clinicaltrails.com	■							
Reclutamiento de personas participantes y aleatorización de las dos condiciones de estudio				■	■	■	■	
Formación y supervisión del terapeuta		■	■	■	■	■	■	■
Evaluación pre-tratamiento				■	■	■		
Recogida de muestras biológicas (cabello) y cuestionarios cognitivos				■	■	■		
Realización de intervenciones psicológicas				■	■	■	■	■
Evaluación post-tratamiento y seguimientos a los 3, 6 y 12 meses				■	■	■	■	■
Análisis de datos					■	■	■	■
Elaboración de comunicaciones para congresos					■	■	■	■
Elaboración y publicación de artículos científicos					■	■	■	■

La fecha prevista de finalización del estudio es diciembre de 2026.

LE RECORDAMOS la documentación a presentar, según aplique, junto con este formulario para la evaluación del proyecto (formato digital)**:**

1. Compromiso del investigador principal y colaboradores (Anexo I)
2. CV de todos los investigadores (principal y colaboradores)
3. Hoja de Información al Participante y Consentimiento Informado (Ver plantilla en la web del CEICA) **o bien** Solicitud de dispensa de HIP/CI y autorización para acceso a los registros (por ej. historia clínica).
4. Memoria económica/ fuente de financiación
5. Autorización para el uso de recursos (Anexo II)
6. Certificado de póliza de seguro (si procede).
7. Certificado marcaje CE y ficha técnica (si es un producto sanitario).
8. Compromiso de uso de datos seudonimizados **SÓLO si cumple el apartado 3.3** (uso de datos de fuente secundaria) (disponible en la web del CEICA)
9. Declaración responsable en caso de estudios observacionales con medicamentos (EOM) sin interés comercial, si procede (ver modelo en la web del CEICA)
10. Si el estudio se realiza en la Universidad se debe presentar la autorización de la Universidad para el tratamiento de datos personales, salvo que se utilicen datos del Sistema Aragonés de Salud.
11. Si el estudio se realiza en algún centro escolar, residencia, asociación, club deportivo, clínica privada o similar, se debe presentar la autorización del responsable del centro para la realización del estudio (formato libre).
