# Supplementary material for: Effectiveness and acceptability of the unified protocol for the transdiagnostic treatment of emotional disorders in people with long COVID-19: Study protocol for a randomized controlled trial
Source: PLoS One. 2026 Feb 17;21(2):e0342908. doi: 10.1371/journal.pone.0342908 (PMC12912558; doi:10.1371/journal.pone.0342908)
Supplement: S2 File — (PDF) [file pone.0342908.s003.pdf]

# S3 – Supporting information 3

**Research project report for ethics committee**

| | |
|---|---|
| **TITLE** | Efficacy and Acceptability of the Unified Protocol for the Transdiagnostic Treatment of Emotional Disorders in People with Long COVID-19 |
| **VERSION AND DATE** | Version 2.0 of May 02, 2025 |
| **STUDY CENTER** | Hospital Royo Villanova |
| **PRINCIPAL INVESTIGATOR OF THE PROJECT** | |
| **NAME AND SURNAME*** | Jorge Osma López |
| **E-MAIL*** | protolocolounificadocovid19@gmail.com |
| **JOB STATION** | Full Professor |
| **SERVICE/DEPARTMENT** | Department of Psychology and Sociology of the University of Zaragoza |
| **CENTRE/ FACULTY-UNIVERSITY/ OTHER** | Faculty of Social and Human Sciences / Teruel Campus |
| **PROMOTOR (Essential for clinical trials and observational studies with drugs)** | |
| **COMPANY IDENTIFICATION / NAME AND SURNAME*** | University of Zaragoza |
| **E-MAIL*** | gesinves@unizar.es |
| **CONTACT OF THE PERSON IN CHARGE OF HANDLING THE REQUEST** *If desired, add the contact details of the person in charge of managing the details of the request* | |
| **NAME AND SURNAME** | Verónica Martínez Borba |
| **E-MAIL** | v.martinez@unizar.es |

The personal data that may appear in this communication will be incorporated into the processing system for which the Aragonese Institute of Health Sciences (IACS) is responsible. The data will be processed for the management and monitoring of the studies evaluated by CEICA . The data will be deleted when the management and/or processing of the request has been responded to and they are no longer necessary. You have the right to access, rectify and delete the data, as well as the other rights granted to you by data protection regulations before the IACS, with registered office at the Centro de Investigación Biomédica de Aragón. Avenida San Juan Bosco, nº 13, 500009, Zaragoza or by requesting it through the email protecciondedatos.iacs@aragon.es.

**GLOSSARY**

- Anonymization: process by which it is no longer possible to establish by reasonable means the link between a piece of data and the subject to which it refers. It is also applicable to the biological sample.
- BIGAN: a healthcare big data platform (managed by the IACS) that allows access to the data of the Aragonese Health System in a pseudonymous way for use in management and research.
- Biobank: public or private, non-profit establishment that houses one or more collections of biological samples of human origin for biomedical research purposes, organized as a technical unit with criteria of quality, order and destination.
- Center: Institution where a study is carried out (hospital, health center, residence, faculty, private clinic, school, etc.). **In case of doubt, reference should be made to the place where the participants come** from (hospital, school, sports club, etc.)
- Confidentiality Commitment: Document that must be signed by students and residents who carry out an activity in the public health system (model established in Order SSI/81/2017).
- Informed consent: manifestation of the free and conscious will validly issued by a capable person, or by his authorized representative, preceded by the appropriate information.
- CEICA Opinion: document that certifies that CEICA has evaluated a research project and that said project complies with the applicable legal standards and ethical criteria.
- Survey: collection of information in physical or digital format, with or without direct interaction with the source subject.
- Interview: collection of information with direct interaction with the source subject, through verbal responses.
- Research team: A group of researchers who jointly carry out a specific project.
- Primary data source: When data are collected directly from the study participant and on the occasion of the study.
- Secondary source of data: When data already collected (and therefore recorded) that were obtained for a purpose other than the study (care, teaching, etc.) are used for the study.
- Research group: A group of researchers with a common trajectory (publications, funding) directed or coordinated by a Principal Investigator, grouped around a research topic and not necessarily by their healthcare or departmental link.
- Participant Information Sheet: Document informing potential participants of the nature of the study, so that they can give their informed consent.
- Intervention (intervention study): any action that is going to be carried out on a person due to their participation in a study (it can be a pharmacological treatment, physiotherapy, an educational, behavioural, psychological intervention).
- Principal investigator: Researcher who leads the project and is responsible for its design, implementation and dissemination of the results. If the study is multicenter, there should be a principal investigator in each center who is responsible for patients, data, and/or samples.
- Biological sample: any biological material of human origin that can be preserved and that can contain information on the characteristic genetic endowment of a person
- Routine clinical practice: Procedures that are carried out for purely healthcare purposes, regardless of whether or not a person participates in a research study.
- Research project: scientific procedure aimed at gathering information and formulating hypotheses about a certain social or scientific phenomenon.
- Sponsor: An individual, company, institution, or organization responsible for initiating, managing, organizing, and funding a study.

- Pseudonymisation: the processing of personal data in such a way that it can no longer be attributed to a data subject without the use of additional information, provided that such additional information is provided separately and is subject to technical and organisational measures designed to ensure that the personal data is not attributed to an identified or identifiable natural person.

| • **SCOPE AND FUNDING OF THE PROJECT** | |
|---|---|
| Is it a multicenter project? Yes ☐ No ☒ | If so, the complete list of centres must be submitted and a commitment from the research team must be filled in **for each centre in Aragon** (see annex I) |
| Do you have specific funding for the study?<br>Yes ☒ No ☐ | In **all cases,** Annex II: authorisation for the use of resources must be completed. In addition, if yes, the budget and the source of funding must be presented. |

| • **CHARACTERISTICS OF THE STUDY** | |
|---|---|
| **2.1 Is it drug research**? Yes ☐ No ☒ | |
| If yes, choose an option:<br><br>1) ☐ This is an observational study regarding drug treatment (EOM)<br>In this case, specify:<br>Prospective data collection Retrospective data collection Cross-sectional data collection ☐ ☐ ☐<br><br>2) ☐ This is an intervention study: clinical trial with drugs<br>In this case, it must be submitted according to the instructions of the AEMPS<br>(https://www.aemps.gob.es/medicamentos-de-uso-humano/investigacionclinica_medicamentos/ensayosclinicos/#n-espanola) | |
| **2.2 Is this research with medical devices or medical devices**? Yes ☐ No ☒ | |
| If yes, choose an option:<br><br>1) ☐ This is an observational study regarding the use of the medical device<br><br>2) ☐ This is an intervention study: clinical trial with medical devices<br>In this case, it must be submitted according to the CEICA SOP for this type of study<br>(https://www.iacs.es/investigacion/comite-de-etica-de-la-investigacion-de-aragon-ceica/) | |
| **2.3 Is it an investigation with invasive procedures?**<br>(Definition: any intervention carried out for research purposes that involves a physical or psychological risk to the participant).<br><br>If so, an insurance policy must be taken out or minimum risk must be justified | Yes ☐ No ☒ |
| **2.4 Does the research include minors or people incapable of giving consent?**<br><br>If so, an information and informed consent document must be submitted addressed to the guardian/legal representative/family member and another addressed to the minor (adapted to their capacity). Review CEICA template. | Yes ☐ No ☒ |

| 2. CHARACTERISTICS OF THE STUDY | |
|---|---|
| **2.5 Are biological samples used in the study?** | Yes ☒ No ☐ |
| If yes, choose one or more options:<br><br>1) ☐ Samples of care surpluses are used with consent for the project<br>The information and consent document (CEICA template) must be submitted<br><br>2) ☐ Samples of care surpluses are used without consent<br>It must be adequately justified in the section on ethical aspects (art. 58.2 Law 14/2007)<br><br>3) ☒ Samples are collected specifically for this study<br>The information and consent document (CEICA template) must be submitted<br><br>4) ☐ A private sample collection is created<br>To create a new collection, submit the necessary documentation for its evaluation and indicate registration number (nº______________) (see CEICA website)<br><br>5) ☐ Samples already collected from a private sample collection are used<br>In this case, identify the collection number and person in charge:______________________<br><br>6) ☐ Samples are requested from an authorized Biobank. In this case, identify the Biobank:_________<br>The application must be submitted to the biobank | |
| **2.6 Is genetic testing performed?** | Yes ☐ No ☒ |
| **2.7 Are embryos, human embryonic cells, human fetal cells or tissues, or human pluripotent cells obtained through cell reprogramming used?**<br>You should contact the IACS or the responsible institution for further authorizations | Yes ☐ No ☒ |

## 3. PROCESSING OF PERSONAL DATA

**Personal data** is considered to be any data (age, sex) or any information (numerical, alphabetical, graphic, acoustic) about an identified or identifiable natural person; any person whose identity can be determined, directly or indirectly (i.e. who has not been irreversibly anonymised at source) is considered identifiable

| **3.1 Is personal data collected or processed in the study?** | Yes ☒ No ☐ |
|---|---|

If yes, check the applicable option:

☒ The informed consent of the interested party is requested
Submit the information and consent document (CEICA template)

☐ Data obtained for another purpose that have been pseudonymised (e.g. medical history, other research, other records) are used in accordance with D.A. 17 of Organic Law 3/2018.

☐ Others. Specify:

Check the **categories** to which the collected data belongs:

☒ **identification data** (Name, address, email, ID, medical record number, telephone, signature, IP, geolocation, image/voice, others)
☒ **Personal data:** date of birth, place of birth, parents' name, place of work, economic data, sex, marital status, children, academic qualifications, others.
☒ **Opinion data**
☒ **particularly sensitive data**: health, ethnicity, religion, political opinion, sexual life or orientation, trade union membership, special educational needs

**3.2 If the data is collected directly from the data subject (primary source), specify the procedure** (e.g. interview, paper survey, email, telephone, web applications, ...)

The data of this project are collected through two ways:

- Online interview: it will be carried out through the Google Meet platform, with an estimated duration of between 60 and 90 minutes. Its main objective will be to verify that the participant meets the inclusion criteria established for the study.
- Online questionnaire: they will be administered through the Qualtrics platform (https://www.qualtrics.com/support/es/survey-platform/getting-started/qualtrics-gdpr-compliance/#About), guaranteeing compliance with data protection regulations. People will receive an email with a link that will allow them to directly access the corresponding questionnaire or survey.
- Paper questionnaires: which are administered by a member of the research team in the face-to-face hair sampling sessions of the experimental group.

**3.3 If they are not collected directly from the data subject (secondary source),** check the option and specify:

☐ Data are collected from an existing registry (e.g. Medical History) **with the consent** of the interested party.

☐ Data from similar research, for which the consent of the data subject was obtained, is reused. Permission must be presented from the person responsible for the data, the consent form with which they were obtained and the commitment to use the pseudonymised data (see on the website).

☐ Data obtained for another purpose and **without consent for research** (e.g., medical history or other record) is used

In this case, indicate:

☐ The researcher (if he or she is a staff member of the centre) has direct access to the medical record. Present permission from the data controller (if it is the medical history, the authorization of the management for access to data for this study must be presented)

☐ The researcher receives the data already pseudonymized Submit the commitment to use the pseudonymized data (see on the website).

Note: **researchers who do not have an employment relationship with the hospital/center** do not have access to the medical record, so WHENEVER this source is used, they must obtain the pseudonymized data.

In all cases, explain: origin of the data, responsible for it

☐ Data from BIGAN

Submit a report from the Biocomputing Unit (link to the application) and the commitment to use the pseudonymised data (see on the website)

**3.4 Once the information and data have been obtained, how is the privacy of the participants guaranteed?**

☐ Only aggregated data (i.e., data that corresponds to groups of people and not to each of those people) is used.

☐ The data is anonymised (the data cannot be associated with an identified or identifiable person because the link to any information identifying the subject has been irreversibly destroyed)

☒ the data is pseudonymised or encoded (direct identifiers are replaced by a code/pseudonym known only to the research team)

Explain how and by whom the measure is taken:

Each participant will receive a numerical code after signing the informed consent. This code will be entered in online and paper assessments to ensure that your identity remains protected.

**3.5 Data retention period:** specify date of destruction (at least month and year):

The data will be kept until the publication of the scientific papers (approximately until 31 December 2030).

It is generally considered appropriate to keep until the date of publication, if not, it must be justified

**3.6 Data processors (do not fill in in the case of anonymous data)**

A processor is understood to be any natural or legal person, public authority, service or other body that processes personal data, other than the controller (investigator).

If a third party (outside the institution) processes project data, it will be necessary to sign a **data processor contract**. A model can be downloaded from https://seguridad.salud.aragon.es/plantillas/

Indicate which persons are going to process the data collected, specifying who will have access to the identification data:

Principal Investigator: Jorge Osma López
Team Member: Verónica Martínez Borba
Team Member: Sara Garcés Arilla
Team Member: Andrés E Rodríguez Márquez

Are all persons authorised to process the data subject to a confidentiality agreement signed with the centre?

Yes ☒ No ☐

**3.7 Will data be transferred to third parties?** Yes ☐ ☒ No

In the case of transfer, the following must be specified:

- The data transferred are: ☐ identified, ☐ pseudonymised, ☐ anonymised

- To whom they are transferred:

- What data is transferred:

- for what purpose:

- Explain how data is pseudonymized or anonymized:

- If there are international transfers: specify company and country (in this case, the participant's express consent must be requested for this transfer)

## PROCESSING OF PERSONAL DATA

| **3.8 Will recordings (audio/video) be made?**<br>Remember that express consent is needed from the interested party to make recordings, this information must be included in the consent document | Yes ☐ No ☒ |
|---|---|

In the case of recording, the following must be specified:

- Where they will be stored, who has access and the security measures to be applied:

- Retention period of the recordings:

- for what purpose:

- If you use computer applications or "cloud" storage, you must indicate who the service provider is and where their legal residence is, as well as the link to their privacy policy:

**3.9 Information security measures: description of the computer systems to be used**

**It is recalled that servers containing personal data must be located in the territory of the EU (RDL 14/2019)**

- System in which the data is to be stored (personal computer, corporate servers, company or external organization, "cloud" service provider, etc.)

The study data will be stored in a file created with the IBM SPSS software, which will be stored on an internal hard drive within the servers of the University of Zaragoza. The identification of the equipment used for this purpose will correspond to Office 26 of the Faculty of Social and Human Sciences of Teruel, with the reference number 259116.

- Applications that are going to be used for data processing (excel, spss, etc.)

For the analysis and entry of quantitative data, the IBM SPSS Statistics statistical package version 22.0 for Windows (IBM Corp., 2013) will be used. The data collected for the study will be identified by a numerical code and only the research staff in the study will have access to them and will be stored on the devices of the University of Zaragoza.

- If online computer applications or "cloud" storage are used, you must indicate who the service provider is and where their legal residence is, as well as the link to their privacy policy.

The questionnaires are collected through the Qualtrics platform (https://www.qualtrics.com/es/nucleo-de-investigacion/encuestas-en-linea/). Which guarantees data security through data encryption, redundancy, continuous network monitoring and single sign-on (SSO). It is also ISO 27.001 certified and authorized by the FedRAM program. The following links detail the aspects related to security and data protection:

- https://www.qualtrics.com/platform/security/
- https://www.qualtrics.com/support/es/survey-platform/getting-started/data-protection-privacy/

### 3.10 Information Security Measures: Devices

- Indicate whether any type of removable device (portable USB, external hard drive, etc.) is to be used and whether they are to be encrypted

The information will be stored on an external hard drive and encrypted through the Veracrypt app.

- In the event that corporate computer systems are not used, indicate if backups are made.

- Indicate the security measures for documents in paper format (custody, access).

The paper documents collected in the study will be kept under lock and key in office 26 of the Faculty of Social and Human Sciences of the University of Zaragoza (location reference 004129). Once the investigation has been completed and the established retention period has expired, the documents will be destroyed by means of a paper shredder or by an authorised confidential document destruction service.

**General recommendations on data use**

- Do not use Wi-Fi networks to transmit sensitive information.
- Use strong passwords and change them regularly.
- Always encrypt sensitive information that is to be sent by email.
- Make sure that the versions of operating systems and applications are always up to date.
- On personal computers, always use antivirus and that it is updated.
- Never open files attached to emails in which we do not identify the sender.
- Do not use social networks to communicate sensitive information.
- The mobile phone is an unsafe device for handling sensitive information, and the antivirus that can be installed offers little protection.
- Use corporate applications whenever possible
- The use of USB or other removable devices is highly discouraged

**4. DESCRIPTION OF THE RESEARCH PROJECT (complete the fields or attach the complete protocol with the equivalent information)**

### 4.1 Tasks of the research team

Briefly explain who is participating in the study, in what capacity and what tasks they are going to perform, as well as their affiliation (current job position). The CV and the signature of all of them must be submitted in Annex I

If the study is multicenter, an Annex I must be submitted for each center

This study will be carried out by a research team made up of four members:

- Osma López, Jorge J., Associate Professor, University of Zaragoza. As principal investigator, he will be responsible for the general coordination of the study, ensuring the correct development of all its phases and the proper execution of the assigned tasks. He will clinically supervise the team and the progress of the study, ensuring compliance with the established protocols. In the initial phase, they will lead and collaborate in the review of the scientific literature and in the preparation of the evaluation protocol, ensuring the methodological soundness of the project. He will also be in charge of supervising the drafting and submission of the documentation necessary for the approval of the study by the Ethics Committees, as well as the registration of the Randomized Controlled Trial (RCT) in clinicaltrials.

  During the intervention process, they will be in charge of the supervision and training of the therapist and staff in training, ensuring the correct application of the psychological intervention. In addition, he will coordinate the correction and coding of data in the study base. Subsequently, he will supervise

the data analysis to draw relevant conclusions. Finally, he will supervise, coordinate and manage the preparation of scientific articles and the presentation of results at conferences.

- Martínez Borba, Verónica., Assistant Professor, University of Zaragoza. As the team's investigator, she will play a variety of roles in evaluating, monitoring, and analyzing the study data. In the initial phase, he will lead and collaborate in the review of the scientific literature. You will be responsible for the evaluation and selection of subjects, ensuring that the inclusion and exclusion criteria are applied correctly. In addition, it will supervise data collection, coordinating the correct implementation of the established evaluation protocols. It will also be responsible for drafting and sending the necessary documentation for the approval of the study by the Ethics Committees. During the intervention, the therapist will be trained in the correct application of the psychological intervention. Its work will extend to the management, analysis and purification of data. Regarding the dissemination of results, it will play an essential role in the writing of scientific articles and in the presentation of the results at conferences.
- Garcés Arilla, Sara., Research staff of the University of Zaragoza. You will manage the data and the collection of biological samples within the study. They will be responsible for the collection of hair samples for cortisol analysis, ensuring their correct handling and storage . It will also administer tests to assess the cognitive status of participants. They will participate in the organization, coding and management of the data in the study database, guaranteeing the integrity and quality of the information collected. They will support statistical analysis, collaborating in the structuring of the data for their correct interpretation. It will also contribute to the correction and purification of the database. Finally, it will participate in the preparation of scientific reports and articles, collaborating in the dissemination of the results obtained.
- Rodríguez Márquez, Andrés E., Research staff at the University of Zaragoza. It will fulfill the function of supporting the evaluation and selection of participants for the project. It will support the registration and management of the database, carry out the initial evaluations and apply the group intervention based on the Unified Protocol. Subsequently, they will collaborate in the analysis of the data obtained, supporting the interpretation of the results and their application in the study. In addition, they will participate in the preparation and writing of scientific articles. It will also support the presentation of results at congresses.
- Del Corral Beamonte, Esther., Bachelor of Medicine and General Surgery, Royo Villanova Hospital. For the last 5 years she has been working in the high-level isolation unit, in the infectious diseases consultation and in the CPCOVID consultation. He has directed 4 projects focused on CPCOVID or COVID-19. Their participation in the project is essential for the recruitment of the participants they visit at their centre.

**4.2 Justification of the study: Background, current status of the topic, relevance** (Cite the bibliographic references in the following section)

The COVID-19 disease has caused one of the largest pandemics in world history, with health, social and economic consequences of great impact. Two and a half years after the World Health Organization (WHO) declared the pandemic, it has been found that 10-20% of patients with COVID-19 do not manage to recover their previous state of health and develop persistent symptoms over time. This condition has been defined by the WHO as post-COVID-19 (COVID-19 CP) or Persistent COVID-19, characterized by the presence of physical, cognitive, and emotional symptoms that can persist for months and even years after acute infection. Among the most prevalent symptoms are fatigue, shortness of breath and cognitive dysfunction, in addition to significant psychological sequelae. Within these, emotional disorders (ETs) are the most commonly reported in this population, including anxiety disorders, depressive disorders, and related disorders. It is estimated that between

10-20% of people with COVID-19 develop CPCOVID (WHO, 2023). In Aragon, estimates are 10% of people diagnosed with COVID-19, which means that a total of approximately 46000 people suffer from CPCOVID (Government of Aragon, 2022).

According to the literature, people affected by COVID have factors associated with an experience of chronic stress: uncertainty about the course of the disease and adaptation to new health conditions and concern about the long-term consequences (Szabo, 2023). In relation to this data, significantly elevated levels of cortisol in hair have been found in people with different chronic diseases (e.g., chronic pain [Jacobsen et al., 2023] and multiple sclerosis [Pereira et al., 2019]) and these elevated levels have been shown to significantly influence the quality of life of those affected (Wosu et al., 2013). On the other hand, elevated cortisol levels have been linked to poorer mnesic function in various medical conditions (Assayag et al., 2017; Saleem et al., 2017). In this regard, researchers who are part of the team of this project have shown that people affected by CPCOVID present subjective memory complaints and that these complaints are significantly related to their emotional symptoms (Llana et al., 2023). In turn, alterations in the hippocampus have been described after infection caused by CPCOVID that are related to alterations in memory and anxious and depressive symptomatology (Zorzo et al., 2023; for a review). This brain region is particularly affected by elevated cortisol levels (Jo.ls et al., 2008). Although there is data that suggests that the emotional and cortisol response, as well as mnesic deficits, could be factors that interact in the definition of the symptoms classically associated with COVID-CP, so far, cortisol levels in response to chronic stress in people with this condition have not been studied.

In addition to the physical and cognitive alterations described above, the pandemic itself and the restrictive measures taken, such as confinement or reduction of social contact, as well as the job instability and economic difficulties experienced, had an impact on the psychological well-being of the general population (Liu et al., 2021). Thus, a meta-analysis study with a sample from nine different countries (n = 146139) reported that the most common symptoms during the COVID-19 pandemic were Emotional Disorders (TEs; nomenclature that groups anxiety, depression, and related disorders; Bullis et al., 2019) (Liu et al., 2021).

In the particular case of people suffering from CPCOVID, the most frequently reported mental health problems were also anxiety disorders (22.2%) and depression (21.1%); (Deer et al., 2021). In turn, people suffering from CPCOVID could be at risk of chronification of their emotional problem, due to having to cope not only with the physical symptoms of the disease, but also with loneliness and stigma related to skepticism about their symptoms, which in turn could affect their social and family life. their work functioning and quality of life (Brown & O'Brien, 2021; Office of National Statistics, 2021). Thus, a reduction in activities of daily living and a decrease in quality of life are observed in people with CPCOVID (de Oliveira Almeida et al., 2022). For all of the above, it is necessary to take a comprehensive approach to these people, with psychological treatments being of great help for the management of emotional distress, functional recovery and improvement in quality of life.

In this line, the implementation of transdiagnostic interventions such as the Unified Protocol for the Transdiagnostic Treatment of ET (PU; Barlow et al., 2011, 2018), could serve the aforementioned purpose. This protocol is based on the principles of Cognitive Behavioral Therapy (CBT) and the main objective of this protocol is to decrease discomfort in response to intense emotions and reduce maladaptive reactions to emotions of any type of valence. For these reasons, PU focuses on showing the adaptive value of all emotions, on identifying and modifying the maladaptive strategies used by the person to avoid or reduce the intensity of emotions and increase tolerance to intense emotions. This protocol highlights that the consequences of emotional avoidance strategies in the medium and long term are very negative for the person, not only because they maintain the problem, but also because they generate interference in the person's day-to-day life, generating

an even more problematic atmosphere. The PU consists of eight modules designed to train five fundamental emotional regulation skills: mindfulness, cognitive flexibility, opposition to maladaptive emotional behaviors, interoceptive exposure, and emotional exposure (Barlow et al., 2018). Its modular structure allows for greater flexibility and adaptability to various clinical problems, facilitating its application in different therapeutic contexts (e.g., Martínez-Borba et al., 2022; Sauer-Zavala et al., 2021). Regarding its clinical utility in improving ETs in the general population, several systematic reviews and meta-analyses have supported its effectiveness (Sakiris & Berle, 2019; Cassiello-Robbins & Carlucci et al., 2021; Longley & Gleiser, 2023). In Spain, its application in a group format within public mental health units has shown its efficacy and cost-effectiveness in the treatment of ETs, consolidating itself as an efficient therapeutic option (Peris-Baquero et al., 2022; Peris-Baquero & Osma, 2023). In addition, its implementation in cost-effective formats, such as group therapy and online interventions, has been shown to be viable and effective (e.g., Reinholt et al., 2017; Schaeuffele et al., 2022).

On the other hand, different studies indicate that high levels of emotional dysregulation are directly associated with different medical disorders (Smith & MacKenzie, 2006; Suls et al., 2005). Thus, a systematic review study reported that PU could be effective in treating emotional symptomatology in a population that presents, in turn, a medical disorder, also implying improvements in medical symptomatology in some studies (Osma et al., 2021). In this regard, emotional regulation has been suggested as a possible transdiagnostic mechanism for mental health in the context of COVID-19 (Volkert et al., 2021). Considering these data, it can be hypothesized that PU could be useful for the improvement of emotional and physical symptoms in people with COVID-CP.

**4.3 Bibliography** (must be referenced in the previous text)


Barlow, D. H., Farchione, T. J., Fairholme, C. P., Ellard, K. K., Boisseau, C. L., Allen, L. B., & Ehrenreich-May, J. T. (2011). Unified protocol for transdiagnostic treatment of emotional disorders: Therapist guide. Oxford University Press.

Barlow, D. H., Farchione, T. J., Sauer-Zavala, S., Latin, H. M., Ellard, K. K., Bullis, J. R., Bentley, K., Boettcher, H., & Cassiello-Robbins, C. (2018). Unified protocol for transdiagnostic treatment of emotional disorders: Therapist guide (2nd ed.). New York, NY: Oxford University Press.

Bullis, J. R., Boettcher, H., Sauer-Zavala, S., Farchione, T. J., & Barlow, D. H. (2019). What is an emotional disorder? A transdiagnostic mechanistic definition with implications for assessment, treatment, and prevention. Clinical Psychology: Science and Practice, 26(2), 1–19. https://doi.org/10.1111/cpsp.12278

Cassiello-Robbins, C., Southward, M. W., Tirpak, J. W., & Sauer-Zavala, S. (2020). A systematic review of Unified Protocol applications with adult populations: Facilitating widespread dissemination via adaptability. Clinical Psychology Review, 78, 101852. https://doi.org/10.1016/j.cpr.2020.101852

Sakiris, N., & Berle, D. (2019). A systematic review and meta-analysis of the Unified Protocol as a transdiagnostic emotion regulation based intervention. Clinical psychology review, 72, 101751. https://doi.org/10.1016/j.cpr.2019.101751

Deer, R. R., Rock, M. A., Vasilevsky, N., Carmody, L., Rando, H., Anzalone, A. J., Basson, M. D., Bennett, T. D., Bergquist, T., Boudreau, E. A., Bramante, C. T., Byrd, J. B., Callahan, T. J., Chan, L. E., Chu, H., Chute, C. G., Coleman, B. D., Davis, H. E., Gagnier, J., Greene, C. S., ... Robinson, P. N. (2021). Characterizing Long

COVID: Deep Phenotype of a Complex Condition. EBioMedicine, 74, 103722. https://doi.org/10.1016/j.ebiom.2021.103722

Joëls, M., Karst, H., DeRijk, R., & de Kloet, E. R. (2008). The coming out of the brain mineralocorticoid receptor. Trends in Neurosciences, 31(1), 1–7. https://doi.org/10.1016/j.tins.2007.10.005

Llana, T., Méndez, M., Garcés-Arilla, S., Hidalgo, V., Méndez-López, M. (2023). Association between olfactory dysfunction and mood disturbances with objective and subjective cognitive deficits in long-COVID. Frontiers in Psychology, 14, 1076743. https://doi.org/10.3389/fpsyg.2023.1076743

Longley, S. L., & Gleiser, T. S. (2023). Efficacy of the Unified Protocol: A systematic review and meta-analysis of randomized controlled trials. Clinical Psychology: Science and Practice, 30(2), 208.

Liu, X., Zhu, M., Zhang, R., Zhang, J., Zhang, C., Liu, P., et al. (2021). Public mental health problems during COVID-19 pandemic: a large-scale meta-analysis of the evidence. *Transl. Psychiatry* 11, 384–310. doi: 10.1038/s41398-021-01501-9

Martínez-Borba, V., Martínez-García, L., Peris-Baquero, Ó., Osma, J., & del Corral-Beamonte, E. (2023). Unified Protocol for the Transdiagnostic Treatment of Emotional Disorders in people with Post COVID-19 condition study protocol for a multiple baseline n-of-1 trial. Frontiers in Psychology, 14, 1160692.

Martínez-Borba, V., Martínez-García, L., Peris-Baquero, Ó., Osma, J., & del Corral-Beamonte, E. (2024). Guiding future research on psychological interventions in people with COVID-19 and post COVID syndrome and comorbid emotional disorders based on a systematic review. Frontiers in Public Health, 11, 1305463.

Martínez-Borba, V., Peris-Baquero, Ó., Martínez-García, L.Osma, J., & del Corral-Beamonte, E. (2024). Unified Protocol Application in Patients With Long COVID-19 Condition in Osma and Farchione (Ed.), Applications of the Unified Protocol in Health Conditions. Oxford University Press. https://global.oup.com/academic/product/applications-of-the-unified-protocol-inhealth-conditions-9780197564295?cc=pt&lang=en&#

Osma, J., Martínez-García, L., Quilez-Orden, A., & Peris-Baquero, Ó., (2021). Unified protocol for the transdiagnostic treatment of emotional disorders in medical conditions: A systematic review. International Journal of Environmental Research and Public Health, 18(10), 5077.

Peris-Baquero, Ó., & Osma, J. (2023). Unified Protocol for the Transdiagnostic Treatment of Emotional Disorders in Group Format in Spain: Results of a Noninferiority Randomized Controlled Trial at 15 Months after Treatment Onset. Depression and Anxiety, 2023.

Schaeuffele, C., Homeyer, S., Perea, L., Scharf, L., Schulz, A., Knaevelsrud, C., ... & Boettcher, J. (2022). The unified protocol as an internet-based intervention for emotional disorders: Randomized controlled trial. PloS One, 17(7), e0270178.

Schaeuffele, C., Meine, L. E., Schulz, A., Weber, M. C., Moser, A., Paersch, C., ... & Kleim, B. (2024). A systematic review and meta-analysis of transdiagnostic cognitive behavioural therapies for emotional disorders. Nature human behaviour, 1-17.

Szabo, S. (2023). The post–COVID stress syndrome: From the three-stage stress response of Hans Selye to COVID-19. Inflammopharmacology, 31, 2799–2806. https://doi.org/10.1007/s10787-023-01179-z

Volkert, J., Taubner, S., Berning, A., Kling, L., Wießner, H., Georg, A. K., et al. (2021). Transdiagnostic mechanisms of mental health during the COVID-19 pandemic on adults and families in Germany: study protocol of a cross-sectional and 1-year longitudinal study. Front. Psychol. 12, 1–11. https://doi.org/10.3389/fpsyg.2021.720104

Zorzo, C., Solares, L., Méndez, M., Méndez-López, M. (2023). Hippocampal alterations after SARS-CoV-2 infection: A systematic review. Behavioural Brain Research, 455,114662. https://doi.org/10.1016/j.bbr.2023.114662

**4.4 Hypothesis** (statement to be proved)

**Hypothesis:**

- *H1 (OE1):* Participants in the experimental group (Psychological Program based on the Unified Protocol) will show a significant reduction in emotional symptoms (anxiety, depression, stress, emotional dysregulation, dimensions of emotional disorders, memory complaints) and a significant improvement in quality of life, compared to the control group.
- *H2 (OE2):* The clinical improvements obtained in the post-program evaluation will be maintained at follow-up at 3, 6 and 12 months after the intervention.
- *H3 (OE3):* High satisfaction rates and high adherence to psychological intervention based on the Unified Protocol will be found.
- *H4 (OE4):* The results of the longitudinal analysis of cortisol and cortisone levels (stress at the endocrine level) will provide crucial insights into how psychological therapy based on the Unified Protocol can contribute to the well-being of people suffering from CPCOVID.

**4.5 Objectives**

**General Objective:**

To evaluate the efficacy and acceptability of the Unified Protocol (PU) in an online group format for the treatment of emotional disorders in 90 adults with CPCOVID condition and diagnosed with emotional disorder, treated at the Aragonese Health Service.

**Specific Objectives:**

- *OE1*: To conduct a randomized clinical trial (RCT) to compare the efficacy of the Unified Protocol in an online group format in improving emotional symptomatology (anxiety, depression, stress, emotional dysregulation, dimensions of emotional disorders), quality of life, and memory complaints in people with CPCOVID versus a control group.
- *OE2:* To explore the stability of the changes obtained after the intervention over time, evaluating their impact at 3, 6 and 12 months after treatment.
- *OE3:* To analyse the acceptability and satisfaction of the participants after receiving the Unified Protocol in an online group format.
- *OE4:* To evaluate the evolution of chronic stress levels through the levels of cortisol and cortisone accumulated in hair before and after the application of the Unified Protocol in group format.

**4.6 Methodology** (all **<u>of the following fields</u>** must be detailed**):**
<u>Study design</u>
<u>Participants</u>: inclusion/exclusion criteria; mode of recruitment (who and how makes initial contact with participants, submit study dissemination material, if any), sample size (and justification), randomization (if applicable)
<u>Sources of information</u>: detailed variables (data to be collected), origin of the data, when and how they are collected, to what period of time they refer.
<u>Procedures</u>: detail in a differentiated way the purely healthcare procedures from those of the research, present surveys or forms that are going to be used (link in the case of online surveys), risk assessment of the experimental procedures and measures to minimise it.
<u>Statistical analysis</u>
<u>Gender consideration:</u> detail the measures taken to ensure that the results of the study can reflect possible sex/gender differences.
<u>Limitations of the study</u>
<u>In the case of biological samples</u>: detail the type and number of samples, how they are collected, where and by whom they are analysed, when they are destroyed (or final destination)

**Design:**
A parallel randomized clinical trial (RCT) will be carried out with two 1:1 conditions; with an experimental group (immediate treatment group) that receives a psychological intervention based on the Unified Protocol and a waiting list control group (with delayed treatment).

**Participants:**
- *Inclusion criteria:*
  - Belong to the autonomous community of Aragon.
  - Be at least 18 years old.
  - Understand Spanish well.
  - SARS-CoV-2 infection documented by PCR, antigen test or serology (anti-N Ac positive).
  - Persistence of physical symptoms beyond 12 weeks after acute SARS-CoV-2 infection.
  - Have a score of 8 or higher on the OASIS Anxiety Scale and/or a score of 7 or higher on the ODSIS Depression Scale.
  - Meet diagnostic criteria for emotional disorder.
  - Have access to the Internet.
  - Signing of the informed consent.
- *Exclusion criteria:*
  - Emotional symptoms pre-existing to acute SARS-CoV-2 infection.
  - Be currently receiving psychological treatment.
  - Have a diagnosis of a serious mental disorder (e.g., personality disorder, bipolar disorder, etc.).
  - Have active suicidal ideation at the time of evaluation.
  - People on treatment with psychotropic drugs should maintain the dose for the duration of the study, unless medically contraindicated.

- *Sample size:* The study will have a total sample size of 90 participants, distributed equally in two groups:
  - 45 people will receive the intervention based on the Unified Protocol in an online group format. In addition, within this group, participants will be invited to provide a hair sample, before and after receiving the psychological program based on the Unified Protocol. These samples allow the levels of cortisol and cortisone, hormones linked to stress, to be measured in order

to analyse possible changes throughout the intervention. In the face-to-face hair collection session, participants will also be administered measures of cognitive functioning.

- The remaining 45 people will not receive immediate psychological intervention, but will remain on a waiting list for approximately 12 weeks. At the end of this period, they will be evaluated again and will receive the intervention based on the Unified Protocol in an online group format.

Using the G*Power software (Faul et al., 2007), and taking into account the two conditions, the 5 evaluation moments (pre-treatment, post-treatment, and follow-ups at 3, 6 and 12 months) and the statistical models that will be applied to analyze the data, we have obtained a total sample size of 74 participants with a statistical power of 90% and an alpha coefficient of 0.05 and an effect size of 0.30. Considering a dropout rate of 20%, a sample size of 45 participants per condition is estimated (Total N = 90).

- *Randomization:* The assignment of participants to experimental and control conditions will be carried out by randomization procedures using the Randomizer software.

**Sources of information:**

Tests administered to determine inclusion criteria (experimental and control group participants):

- **Structured interview for anxiety disorders and related disorders, according to the DSM-5 (ADIS-5; Brown & Barlow, 2014):** Structured interview evaluating the DSM-5 criteria for anxiety, mood, and related disorders.

Tests administered online through the qualtrics platform (participants of the experimental and control group):

- **Sociodemographic and medical history questionnaire (ad hoc):** gender, age, place of residence (zip code), marital status, employment status (profession), socioeconomic status, weight, height, menstrual status, sleep habits, lifestyle habits (tobacco, alcohol, physical activity, diet), SARS CoV 2 vaccination (Yes/No prior to infection, number of doses and date of these, brand of vaccine).
- **Long COVID Pre Assessment Questionnaire (National Health Service, 2021):** a questionnaire that assesses different physical symptoms present in CPCOVID.
- **General Severity and Interference Scale for Depression (ODSIS; Osma et al., 2019):** frequency, intensity, severity, and interference of depressive symptomatology.
- **General Severity and Interference Scale for Anxiety (OASIS; Osma et al., 2019):** frequency, intensity, severity, and interference of anxious symptomatology.
- **Multidimensional Inventory for Emotional Disorders (MEDI; Osma et al., 2021):** transdiagnostic profile of Emotional Disorders, composed of nine dimensions: neurotic temperament, positive temperament, depressed mood, somatic anxiety, arousal, social anxiety, intrusive cognitions, traumatic re-experience, and avoidance
- **Emotional Regulation Difficulties Scale (DERS; Hervás & Jodar, 2008):** difficulties in emotional regulation through 5 subscales (lack of control, rejection, interference, inattention, and emotional confusion).
- **Stress Tolerance Scale (DTS; Sandín et al., 2017):** evaluation through 15 items of tolerance to discomfort. It assesses the following dimensions: 1) Perceived ability to tolerate emotional distress; 2) Subjective assessment of discomfort; 3) Attention absorbed by negative emotions; 4) Regulatory efforts to alleviate discomfort.

- **Daily Life Memory Failures (MFE) Questionnaire; Montejo et al., 2012):** evaluation of forgetfulness in everyday life.
- **EuroQol-5D (Badia et al., 1999):** self-perceived health status.
- **Stress Perception Scale (PSS; Remor & Carrobles, 2001):** perception of the stress experienced in the last month.
- **Treatment Satisfaction Questionnaire (STQ; ad hoc):** satisfaction with the program received, based on its perceived quality, adequacy of their expectations, their recommendation of the program to loved ones, the usefulness of the techniques learned, and their overall satisfaction with the program and its format. It is administered only after administration of the program (T2).
- **Evaluation questionnaire for the PU modules (ad hoc):** a general question that assesses the usefulness of the programme in improving emotional regulation and six specific questions that separately assess the usefulness of each of the techniques worked on in the different PU modules to better regulate emotions. The response scale is Likert type and ranges from 0 (not at all) to 10 (a lot). It is administered only after administration of the program (T2).

Tests administered in person (participants in the experimental group):

- **Montreal Cognitive Assessment (MoCA; Nasreddine et al., 2005):** brief neuropsychological test designed to assess mild cognitive impairment. It assesses multiple cognitive domains, including visuospatial skills, memory, working memory, attention, concentration, language, executive functions, and orientation.
- **Digit and symbol test: subtest of the Wechsler Adult Intelligence Scale-III (WAIS-III; Wechsler, 1997):** the digit subtest analyzes attention span, short-term memory, and working memory, by repeating numerical sequences in direct and reverse order, which allows the ability to retain and manipulate auditory information immediately. For its part, the symbol subtest (Keys) assesses processing speed, sustained attention, visual-motor coordination and immediate associative learning, asking the person to relate numbers to symbols following a key and in a limited time.
- **Digit subtest; subtest of the Wechsler Adult Intelligence Scale-IV (WAIS-IV; Wechsler, 2008):** The direct order digits task of this subtest assesses verbal short-term memory and consists of a numerical recall task that measures the mechanical repetition of a sequence of numbers.
- Questionnaire **to collect relevant information on hair characteristics and treatments of the participants (ad hoc):** this instrument includes questions related to the current state of the hair, suchas whether it is treated, if they have received chemical treatments, and if they have hair loss problems , among other aspects.

**Procedures:**

The recruitment of participants will be carried out through the Royo Villanova Hospital in Zaragoza. Dr. Esther del Corral Beamonte will offer patients seen in person at her office the possibility of participating in the study. The inclusion criteria that Dr. Esther del Corral Beamonte will take into account are that the possible participants: reside in the Autonomous Community of Aragon, are of legal age, understand Spanish, have documented SARS-CoV-2 infection and diagnosis of persistent COVID-19, have access to the Internet and are willing to receive the psychological program in an online group format. People who meet these initial inclusion criteria will receive an informative leaflet to access the Qualtrics online platform. Those participants who are interested in participating in the study will have to enter the link or QR code shown on the leaflet to access the Qualtrics online platform where they will find the information document and informed consent (attached). After signing the online informed consent, a random numerical code will appear to each participant. Once the

consent has been signed, the psychologist in charge of carrying out the psychological program (Andrés Rodríguez Márquez) will contact the participants by email and telephone to arrange an online session via video call. This online and individual session will last approximately 60 to 90 minutes, and will serve to carry out the clinical interview where the inclusion and exclusion criteria to participate in the study will be confirmed (ODSIS and OASIS score, meeting the diagnostic criteria for emotional disorder, not having a serious mental disorder or active suicidal ideation). Participants who are excluded from the study will be informed of possible intervention alternatives that are more suited to their current problems. After the interview, a Qualtrics link will be emailed to complete the pre-intervention assessment protocol (T1). Subsequently, they will be informed by email of the condition to which they have been randomly assigned (experimental group or waiting list control group).

Participants in the experimental group will be invited to participate in the collection of hair samples, to determine changes in cortisol and cortisone levels accumulated at three measurement points (pre-treatment; three months after the end of treatment; and 12 months after the end of treatment). This procedure will be carried out individually and in person with Sara Garcés Arilla. Likewise, in this face-to-face session, the selected people will complete the MoCA, the WAIS III subtest (digits and symbols), the WAIS IV subtest (digits) and the ad hoc questionnaire on hair issues.

After responding to the pre-program evaluation (and providing the hair sample in the case of the experimental group), participants will receive the psychological intervention based on the Unified Protocol. This procedure will be different depending on the condition to which the person has been assigned:

Experimental group-Psychological intervention based on Unified Protocol (PU) in online group format: people assigned to this group will receive a brief written support manual with the contents to be worked on in the psychological intervention and will attend 12 sessions of the psychological intervention based on the PU in online group format, through the Google Meet platform. The sessions will be held once a week, with a duration of 2 hours each. The structure of the psychological intervention and content of the sessions you will receive is as follows:

- Module 1 (1 session): goal setting and motivation for change.
- Module 2 (2 sessions): understanding your emotions and analysis of emotions.
- Module 3 (2 sessions): emotional awareness.
- Module 4 (2 sessions): cognitive flexibility.
- Module 5 (1 session): opposing emotional behaviors.
- Module 6 (1 session): interoceptive presentations.
- Module 7 (2 session): emotional exposures.
- Module 8 (1 session) relapse prevention.

Once the psychological intervention is finished, people will be asked to access a Qualtrics link to fill out the post-intervention evaluation (T2), which lasts approximately 25 to 30 minutes. Likewise, three follow-up sessions will be carried out, at 3 (T3), 6 (T4) and 12 (T5) months after the end of the intervention.

Control group-Waiting list: people assigned to this condition will not receive psychological intervention immediately. Instead, after the pre-program evaluation, they will remain on the waiting list for 12 weeks. Once this waiting period is over, they will undergo the post-program evaluation through Qualtrics and receive the PU-based psychological intervention in an online group format. The duration, contents of the sessions and procedures will be exactly the same as those described in the condition of the experimental group "Psychological intervention based on PU in online group format".

Since the program received by participants in the experimental group consists of synchronously attending a psychological program provided in a group format, it is not possible to perform the blinding recommended in RCTs. Thus, both the participants and the psychologist in charge of applying the psychological program will be aware of the condition to which the participants have been assigned. However, to reduce the possibility of bias in the intervention, participants will be randomly assigned to each of the conditions (avoids choice bias) and part of the evaluation will be carried out through an online platform and self-reported questionnaires (evaluator bias is avoided).

**Statistical analysis:**

Descriptive statistics, Pearson's r-correlations, multiple linear regressions, and linear mixed-effects models using the lm4 package (version lme4_1.1-13; Bates et al., 2015) for R statistical software (version 4.1.0; R Core Team, 2021) and SPSS v25.0 (IBM Corp, 2017). Taking into account the nature of the variables to be included in the models, several linear mixed-effects models will be used for each dependent variable to be analysed (each of the different questionnaires used in the pre- and post-intervention measurements). For each of these models, Time (intrasubject variable; T1 <pre-treatment> vs. T2 <post-treatment> vs. T3 <3 months> vs. T4 <6 months> vs. T5 <12 months>) and the Experimental Group (intersubject variable; Experimental vs. Waiting list) will be taken into account as fixed effects of the model. Random slopes for participants will also be taken into account, which will be included as random effects of the model. The models will have the following structure: [Dependent Variable ~ Time × Experimental Group + (1|Participant)].
For hair samples, repeated measures ANOVA will be performed with a factorial design. A correlational approach will be established to determine associations between cortisol and other dependent variables of an emotional type and memory complaints.

**Consideration of gender perspective:**

It should be noted that the incidence of CPCOVID and TEs is higher in the case of women. In this sense, the results of a study carried out in Spain with 341 people with CPCOVID (Mateu et al., 2023), found that 69.8% were women (prevalences similar to those obtained in other studies such as Nielsen et al., 2022), and that being a woman, together with having a history of headache, tachycardia, fatigue, neurocognitive complaints or dyspnea predicted the development of CPCOVID. and a lower probability of recovering from CPCOVID (Mateu et al., 2023). Similarly, the prevalence of ETs has a higher incidence in women, in fact, the diagnosis of depressive disorders is twice as prevalent as its diagnosis in women (Santomauro et al., 2021). Suffering from CPCOVID and also ET has been associated with significant impairments in quality of life (Malick et al., 2021), decreases in functional status in 94% of cases (Nielsen et al., 2022) and more than 56% of sick leave (Nielsen et al., 2022).

This study aims to address social and gender inequalities in health with the inclusion of the gender variable (along with other sociodemographic and clinical variables) in all statistical analyses carried out and, in this way, contribute to the advancement of knowledge about gender differences in CPCOVID and in the psychopathology of ETs and in the cost-effectiveness. adherence and acceptability of psychological interventions for these disorders. For the preparation of all scientific papers, the gender and age variables of all participants will be taken into account. This knowledge will be used to design future adaptations of psychological interventions according to gender (personalization of treatments) and thus contribute to reducing gender inequalities in mental health.

**Limitations of the study:**

Throughout the implementation of the study, some limitations may arise that must be taken into account in the interpretation of the results. Firstly, one of the main obstacles identified is the possible increase in workload for the collaborating professionals in charge of facilitating the sample. To minimize this overload, assessmentswill preferably be conducted online using the Qualtrics platform, allowing for automatic and secure data storage. Second, there could be loss of information during evaluations. However, the digital data collection format is set up to avoid omissions, ensuring that all questions are completed before submitting forms. Another limitation to consider is the possibility of finding anomalous values in the analysis of capillary samples, which could affect the interpretation of cortisol levels. To this end, a specific questionnaire on hair treatments is incorporated and cortisone levels will also be measured , in order to increase the interpretative validity of the results. Finally, to guarantee fidelity when applying the psychological intervention based on the Unified Protocol, the therapist will receive a complementary training session, as well as continuous supervision sessions , aimed at resolving doubts, motivation and the monitoring of fidelity in the application of the protocol.

**Biological samples:**

The procedure for taking biological samples will consist of taking 8 to 10 hairs from the back of the head. To obtain such a sample, the hairs about three centimeters long from the back of the head will be carefully cut, cut as close as possible to the scalp. The sample will be analyzed by the Kirschbaum laboratory of the Technische Universität Dresden, located in Germany, specialized and with a seal of quality (https://dresden-lab-service.com/quality-control/) to determine the level of cortisol and cortisone accumulated during the last three months. The samples will be stored in aluminium envelopes, identified by an anonymous code to identify the sample, inside a zip bag. This type of sample does not require refrigeration, so they will later be stored in a cabinet in the psychobiology laboratory of the Teruel Campus of the University of Zaragoza (location reference 004129), to which only the personnel in charge of the research will have access. Once the project is completed, samples or any excess material will be destroyed. It should be noted that the analysis is not for diagnostic purposes of any physical or psychological disease.

**4.7 Ethical aspects** (risk/benefit balance, **justification in case of requesting exemption from informed consent** , care implications, implications for the participant or his/her family, compensation to participants, insurance policy).

All people who meet the inclusion criteria and participate in the trial will sign the Data Protection document so that they have an idea of who is going to use the results of this research and for what purpose. They will also be informed about what the psychological intervention consists of, as well as its duration and the phases of the study. To this end, they will be provided with the information sheet explaining the treatment, and its respective phases, as well as the document corresponding to the Informed Consent.
With regard to the confidentiality, processing, communication and transfer of the personal data of all participating subjects, it will be in accordance with the provisions of the Declaration of Helsinki (Seoul, 2008), Law 14/2007 on Biomedical Research, Organic Law 3/2018, of 5 December, on the Protection of Personal Data and the Guarantee of Digital Rights (LOPD GDD). As of May 25, 2018, the new legislation on personal data in the EU is fully applicable, specifically Regulation (EU) 2016/679 of the European Parliament and of the Council of April 27, 2016 on Data Protection (GDPR). In accordance with the provisions of the aforementioned legislation, participants may exercise their rights of access, modification, opposition and cancellation of data, for which they must contact the principal investigator in charge of the study. The personal information collected for the study will be replaced by numerical codes and the sociodemographic data will be stored separately and will only be accessible by the researchers responsible for the storage and processing of the data, always protecting the right to privacy.

Information on psychosocial and cognitive measures will be collected through the Qualtrics platform. This particular platform is GDPR and CCPA compliant. Qualtrics is committed to keeping customer data secure and providing capabilities to help customers comply with any privacy and data security regulations to which they may be subject.

Research projects where a psychological intervention is applied often include a control group that does not receive the psychological intervention in a delayed manner, but receives it a few weeks after the experimental group begins the program. This makes it possible to determine which of the improvements observed in the experimental group are really due to the psychological program and which could be explained by other factors such as the passage of time. For ethical reasons, and with the aim that all participants can benefit from the psychological program, it is proposed that the control group remain on the waiting list for the 12 weeks that the psychological program lasts, being able to carry out the intervention after this time. For this reason, physiological measurements to analyze cortisol levels in hair cannot be collected in the control group. Cortisol hair tests provide an indicator of cortisol levels over the past 3 months. For this reason, it is necessary to know the cortisol levels at the post-program moment you have to wait three months (this measurement is included in the follow-up at three months and not the post-program moment). In order for the control group to benefit from the program as soon as possible, it has been decided not to collect cortisol measurements in this group. At the same time, with the aim of reducing the burden of conducting extensive psychological assessments, especially when participants do not benefit from the psychological program, participants in the control group do not perform the face-to-face assessment where measures of intelligence and cognitive impairment are administered.

**4.8 Timeline and work plan**:

- Development stages, duration, estimated start and end dates (indicate at least month and year).
- Places where the project is planned to be carried out, facilities to be used.

| | 2025 | | | | 2026 | | | |
|---|---|---|---|---|---|---|---|---|
| **Tasks** | **1** | **2** | **3** | **4** | **1** | **2** | **3** | **4** |
| *Preparation, documentation and application for approval of the study by the Clinical Research Ethics Committee of Aragon* | | | | | | | | |
| *Registering the RCT in clinicaltrails.com* | | | | | | | | |
| *Recruitment of participants and randomization of the two study conditions* | | | | | | | | |
| *Therapist training and supervision* | | | | | | | | |
| *Pre-treatment evaluation* | | | | | | | | |
| *Collection of biological samples (hair) and cognitive questionnaires* | | | | | | | | |
| *Carrying out psychological interventions* | | | | | | | | |
| *Post-treatment evaluation and follow-up at 3, 6 and 12 months* | | | | | | | | |
| *Data analysis* | | | | | | | | |
| *Preparation of communications for congresses* | | | | | | | | |
| *Preparation and publication of scientific articles* | | | | | | | | |

The expected completion date of the study is December 2026.

**WE remind you of the documentation to be submitted, as applicable, along with this form for the evaluation of the project** (digital format):

1. Commitment of the principal investigator and collaborators (Annex I)
2. CVs of all researchers (principal and collaborators)
3. Participant Information and Informed Consent Sheet (See template on the CEICA website) **or** Request for dispensation from HIP/CI and authorization for access to records (e.g. medical history).
4. Financial report/source of funding
5. Authorization for the use of resources (Annex II)
6. Insurance policy certificate (if applicable).
7. CE marking certificate and technical data sheet (if it is a medical device).
8. Commitment to use pseudonymised data **ONLY if it complies with section 3.3** (use of secondary source data) (available on the CEICA website)
9. Responsible declaration in case of observational studies with medicinal products (EOM) with no commercial interest, if applicable (see model on the CEICA website)
10. If the study is carried out at the University, the University's authorisation for the processing of personal data must be presented, unless data from the Aragonese Health System is used.
11. If the study is carried out in a school, residence, association, sports club, private clinic or similar, the authorisation of the person in charge of the centre must be presented for the study to be carried out (free format).
